# Antibody–drug nanoparticle induces synergistic treatment efficacies in HER2 positive breast cancer cells

**DOI:** 10.1038/s41598-021-86762-6

**Published:** 2021-04-01

**Authors:** Muhammad Raisul Abedin, Kaitlyne Powers, Rachel Aiardo, Dibbya Barua, Sutapa Barua

**Affiliations:** 1 Department of Chemical and Biochemical Engineering, Missouri University of Science and Technology, 110 Bertelsmeyer Hall, 1101 N. State Street, Rolla, MO 65409-1230 USA; 2 Department of Electrical and Computer Engineering, Missouri University of Science and Technology, Rolla, MO 65409 USA

**Keywords:** Biotechnology, Biomaterials, Drug delivery

## Abstract

Chemotherapeutic drugs suffer from non-specific binding, undesired toxicity, and poor blood circulation which contribute to poor therapeutic efficacy. In this study, antibody–drug nanoparticles (ADNs) are engineered by synthesizing pure anti-cancer drug nanorods (NRs) in the core of nanoparticles with a therapeutic monoclonal antibody, Trastuzumab on the surface of NRs for specific targeting and synergistic treatments of human epidermal growth factor receptor 2 (HER2) positive breast cancer cells. ADNs were designed by first synthesizing ~ 95 nm diameter × ~ 500 nm long paclitaxel (PTX) NRs using the nanoprecipitation method. The surface of PTXNRs was functionalized at 2′ OH nucleophilic site using carbonyldiimidazole and conjugated to TTZ through the lysine residue interaction forming PTXNR-TTZ conjugates (ADNs). The size, shape, and surface charge of ADNs were characterized using scanning electron microscopy (SEM), SEM, and zeta potential, respectively. Using fluorophore labeling and response surface analysis, the percentage conjugation efficiency was found > 95% with a PTX to TTZ mass ratio of 4 (molar ratio ≈ 682). In vitro therapeutic efficiency of PTXNR-TTZ was evaluated in two HER2 positive breast cancer cell lines: BT-474 and SK-BR-3, and a HER2 negative MDA-MB-231 breast cancer cell using MTT assay. PTXNR-TTZ inhibited > 80% of BT-474 and SK-BR-3 cells at a higher efficiency than individual PTX and TTZ treatments alone after 72 h. A combination index analysis indicated a synergistic combination of PTXNR-TTZ compared with the doses of single-drug treatment. Relatively lower cytotoxicity was observed in MCF-10A human breast epithelial cell control. The molecular mechanisms of PTXNR-TTZ were investigated using cell cycle and Western blot analyses. The cell cycle analysis showed PTXNR-TTZ arrested > 80% of BT-474 breast cancer cells in the G2/M phase, while > 70% of untreated cells were found in the G0/G1 phase indicating that G2/M arrest induced apoptosis. A similar percentage of G2/M arrested cells was found to induce caspase-dependent apoptosis in PTXNR-TTZ treated BT-474 cells as revealed using Western blot analysis. PTXNR-TTZ treated BT-474 cells showed ~ 1.3, 1.4, and 1.6-fold higher expressions of cleaved caspase-9, cytochrome C, and cleaved caspase-3, respectively than untreated cells, indicating up-regulation of caspase-dependent activation of apoptotic pathways. The PTXNR-TTZ ADN represents a novel nanoparticle design that holds promise for targeted and efficient anti-cancer therapy by selective targeting and cancer cell death via apoptosis and mitotic cell cycle arrest.

## Introduction

Engineering nanoscale drug delivery systems have been studied extensively because of several advantages including high drug payloads^1–5^, improved drug release^6,7^, enhanced bioavailability^8,9^, and increased multivalence effects through receptor-ligand interactions^10–13^. The use of a ligand allows direct delivery of the cytotoxic agent to target cells, however, the clinical success of targeted antibody–drug conjugates is limited due to poor bioavailability and low therapeutic efficacy^14–16^. Improvements in drug design in combination with active targeting are shown to enhance cellular uptake, targeting, and higher therapeutic efficacy which have led to the design of antibody–drug nanoparticles (ADNs). ADNs are comprised of an anticancer drug core nanoparticle conjugated with a therapeutic monoclonal antibody on the nanoparticle surface for targeted delivery and enhanced therapeutic efficacy. Trastuzumab (TTZ) is used to treat early-stage human epidermal growth factor receptor 2 (HER2) positive breast cancer as the first line of treatment. However, the complete response of the treatment is not achieved in 20–60% cases of the HER2 positive breast cancer patients^17^. The TTZ treatment is associated with the risk of therapeutic resistance and tumor recurrence after a certain period of the treatment. A lower risk of tumor recurrence and a better outcome can be expected when TTZ is administered with another chemotherapeutic drug in combination^18,19^. Till 2013, four Phase III clinical trials involving more than 8000 patients showed that the risk of recurrence was decreased by 50% when TTZ was administered with or after chemotherapy in combination^20–24^. The American Society of Clinical Oncology (ASCO) guideline recommended anti-HER2 therapy in combination with taxane as the first line of treatment for metastatic HER2 positive breast cancer^25^.

Combination therapy using paclitaxel (PTX) and TTZ for adjuvant HER2 positive breast cancer showed promising long-term clinical outcomes in recent years. In 2015, after a median follow up period of 4 yr, adjuvant PTX and TTZ combination treatment trials reported that the 3 yr disease-free survival rate was 98.7% in HER2 positive breast cancer patients^26^. In 2019, after 7 yr follow up period, the disease-free survival rate was 93% with an overall survival rate (OS) of 95%^27^. While the combination therapy of PTX and TTZ are promising, the following challenges still have to overcome in terms of synergy between the drugs, toxicity, and therapeutic efficacy^28^. The current clinically used intratumoral concentration of PTX (1–9 μM) is shown to be insufficient for breast cancer treatment^29^. Moreover, PTX is poorly soluble in the aqueous phase causing a great limitation and trade-off in terms of toxicity in intravenous administration. The high concentration of TTZ used in clinical trials has always been associated with the risk of severe cardiac arrest^26–28^.

To overcome the limitations of toxicity and off-site targeting, nanoparticles have been engineered with advanced chemical composition^14–16,30^, surface functionalization^31–35^, and geometric modification^35–38^. Nanoparticles made of human serum albumin (Abraxane) have been used for the delivery of PTX to breast cancer cells^39^. Another example of a nanoparticle-based drug delivery system is liposome-based doxorubicin (Doxil) for the treatment of ovarian cancer^40,41^ and breast cancer^42^. However, the efficiency of these nanoparticles is controversial due to the lack of active targeting and controlled drug delivery. Currently, a large number of nanoparticles such as liposomes^43^, polymers^44^, micelles^45^, solid lipid nanoparticles^46^, and other organic and inorganic nanoparticles have been explored to improve the therapeutic efficiency by active targeting of cancer cells through the design of nanoparticle size, shape and surface charge.

Nanoparticle size in the range of 50–500 nm is desirable for enhanced blood circulation, tissue penetration, and cellular interaction^36,47,48^. Size less than 10 nm is cleared by renal excretion, while larger particles are cleared by phagocytosis^11,12,49,50^. Non-spherical nanoparticles have been reported to be less clearance by macrophages than spherical nanoparticles as dictated by the contact angle of nanoparticles with the macrophage membrane^51^. Nanoparticle also leads to a dramatic increase in surface area for surface functionalization with targeting ligands such as monoclonal antibodies, affibodies, peptides, and aptamers. For example, binding of rod-shaped nanoparticles can be high if the particle aligns with its major axis parallel to the cell membrane increasing the multivalence effects of receptor-ligand interactions^10–13^.

In this study, we synthesized PTX nanorods (PTXNRs) of (96.8 ± 33) × (503.4 ± 210) nm using the nanoprecipitation method. TTZ was used as a HER2 targeting antibody for specific PTX delivery to HER2 positive breast cancer cells (BT-474 and SK-BR-3). The surface of PTXNRs was conjugated via carbonyldiimidazole (CDI), a highly reactive crosslinker to form an active N-acyl imidazole group capable of coupling with amine-containing TTZ and formed a stable amide linkage. Flow cytometry analysis of a fluorophore-labeled PTXNR-TTZ and response surface analysis confirmed >95% TTZ conjugation efficiency. A combination approach of PTXNR-TTZ ADNs was explored in exploiting the therapeutic efficacy of ADNs towards HER2 positive breast cancer cells. It is hypothesized that a combination system using PTXNR-TTZ ADNs enhances therapeutic payloads and treatment efficiency by selective targeting of HER2 positive breast cancer cells.

## Materials and methods

### Synthesis of PTXNRs

PTXNRs were prepared using the nanoprecipitation method (SI Fig. 1)^10,52,53^. Briefly, PTX powder (> 99.5%, Alfa Aesar) was dissolved in 5 ml of ethanol at a concentration of 3 mg/ml. The solution was homogenized at 1000 rpm, sonicated, and injected into 20 ml deionized (DI) water at a flow rate of 2 ml/min to form PTXNRs. PTXNRs were washed three times using DI water by centrifuging at 18,000 rcf for 2 h, suspended and lyophilized (Labconco) overnight to obtain dried particles.

**Figure 1:**
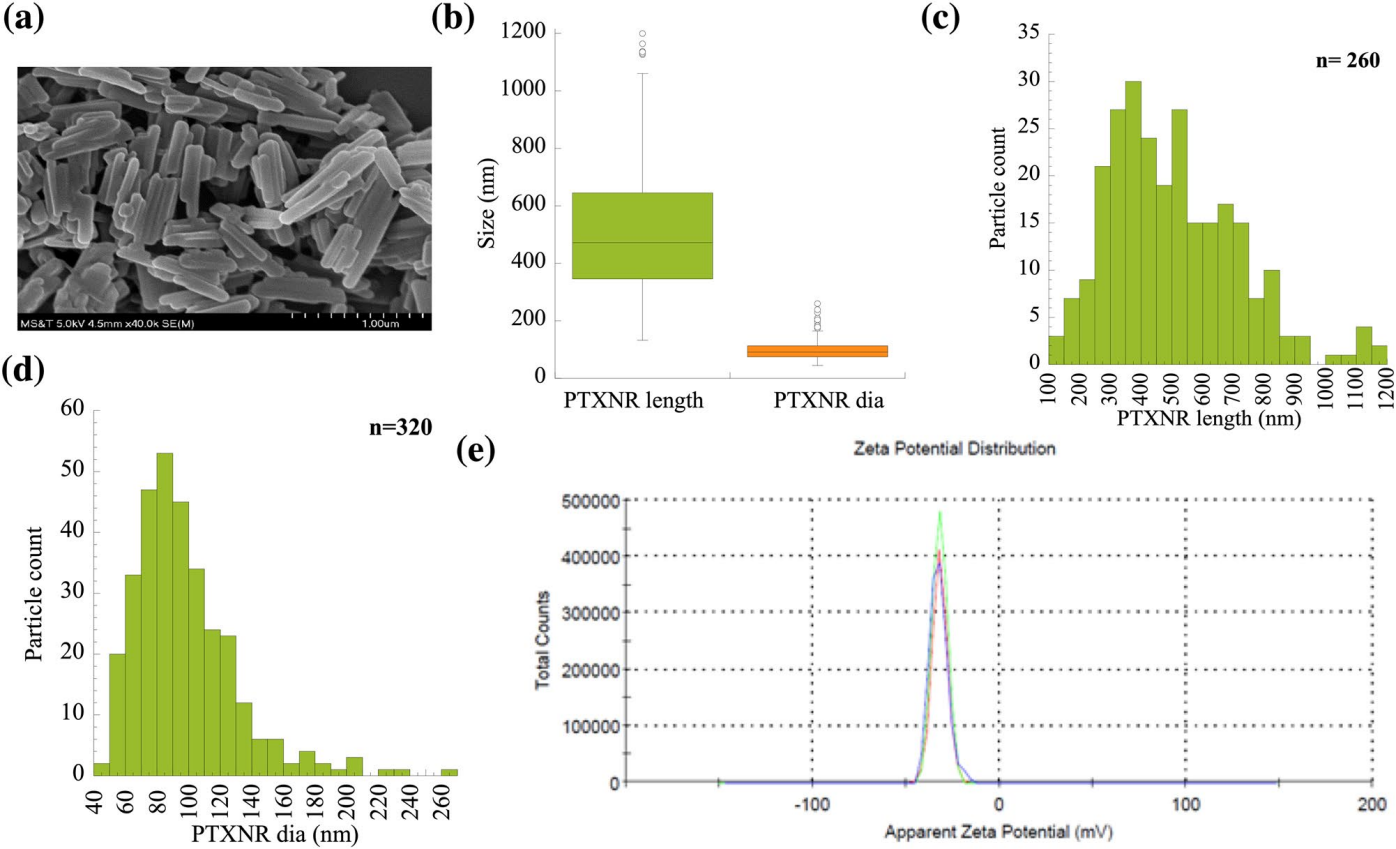
Characterization of PTXNRs: (**a**) Scanning electron microscopy (SEM) image of PTXNRs. (**b**) Particle size: Percentile plots of the PTXNR-TTZ particle length and diameter were analyzed using ImageJ/ Fiji software. (**c**) Size distribution of PTXNR particle length. (**d**) Size distribution of PTXNR particle diameter as calculated analyzing SEM images. The number ‘n’ denotes the particle population count for the analysis; and (**e**) Zeta potential (ξ) of PTXNR is -32.6 ± 4.8 mV in water. Three colors indicate three different experiments.

### Characterization of PTXNR

The size and shape of PTXNRs were analyzed using a scanning electron microscope (Hitachi S4700 FESEM). The images were obtained at 10.0 kV accelerating voltage with 4.5 mm working distance and 40,000× magnification. The size distribution of NRs was analyzed using Fiji image processing software (Image J; Win 64 Java 1.8.0). The surface charge of PTXNRs was measured in DI water and phosphate-buffered saline (PBS) using zeta potential (Nano series Zetasizer, Malvern).

### Activation of 2′ OH nucleophilic site of PTXNR

TTZ (Genentech) was conjugated with the 2′ OH nucleophilic site group of PTX using the CDI activation reaction. Briefly, 1 mg of dry PTXNRs was suspended in 1 ml of PBS. 5 mg CDI was added directly to the NR suspension with a continuous stirring speed of 600 rpm. The reaction was carried out at 4 °C for 20 min to form the amine-reactive PTX-carbamate (PTXNR-CDI). The PTXNRs-CDI were centrifuged at 16,000× *g* for 30 min and washed five times using DI water to remove the unreacted CDI or imidazole. The particles were lyophilized for subsequent TTZ conjugation reactions.

### ^1^H-NMR analysis of activated PTXNR

To confirm the linkage of the activated carbamate group at the 2′ OH site of PTXNR, the ^1^H-NMR experiment was carried out. In carrying out the experiment, 2 mg of each unmodified PTXNR and surface functionalized PTXNR particles were dissolved in 600 µl of chloroform-d solvent (Alfa Aesar). The solvent was used as the internal reference to determine chemical shifts (δ) in ppm. ^1^H-NMR spectra were then recorded using Bruker advanced III 400 MHz Liquid-State NMR instrument at R.T.

### Conjugation of TTZ with activated PTXNR through the lysine residue interaction

The CDI activated PTXNRs were subsequently reacted with the ε-amino group of lysine residues of TTZ (pKa 10.53) for subsequent conjugation^54^. 100 µg of TTZ powder was dissolved in 100 µl carbonate buffer at pH 9.3–9.5 and added to the CDI activated 100 µl of 1 mg PTXNR-CDI particle suspension. The reaction was allowed to proceed for 48 h at room temperature (~ 22 °C). The resulting TTZ conjugated PTXNRs (PTXNR-TTZ) were centrifuged at 16,000× g for 25 min and washed using carbonate buffer at pH 9.3–9.5 (3 × 1 ml). The supernatant was collected after each washing for quantifying the unbound TTZ. The concentrate was collected by centrifuging the membrane filter at 1000× *g* for 2 min. The concentrated PTXNR-TTZ particles were finally re-suspended in 300 µl of PBS (pH 7.5). The amount of PTXNR in PTXNR-TTZ was quantified by measuring absorbance at 230 nm (BioTek Synergy 2; BioTek, Winooski, VT, USA) using a PTX calibration curve (SI Fig. 2). The amount of unbound antibody was quantified using a BCA protein Assay (Pierce Biotechnology, Rockford, IL, USA) and a TTZ calibration curve (SI Fig. 3). The size and shape of PTXNR-TTZ particles were investigated using SEM (10.0 kV; accelerating voltage with 5.6 mm working distance and 20,000× magnification). The surface charge of PTXNR-TTZ was measured in DI water and PBS using a Nano series Zetasizer (Malvern).

**Figure 2:**
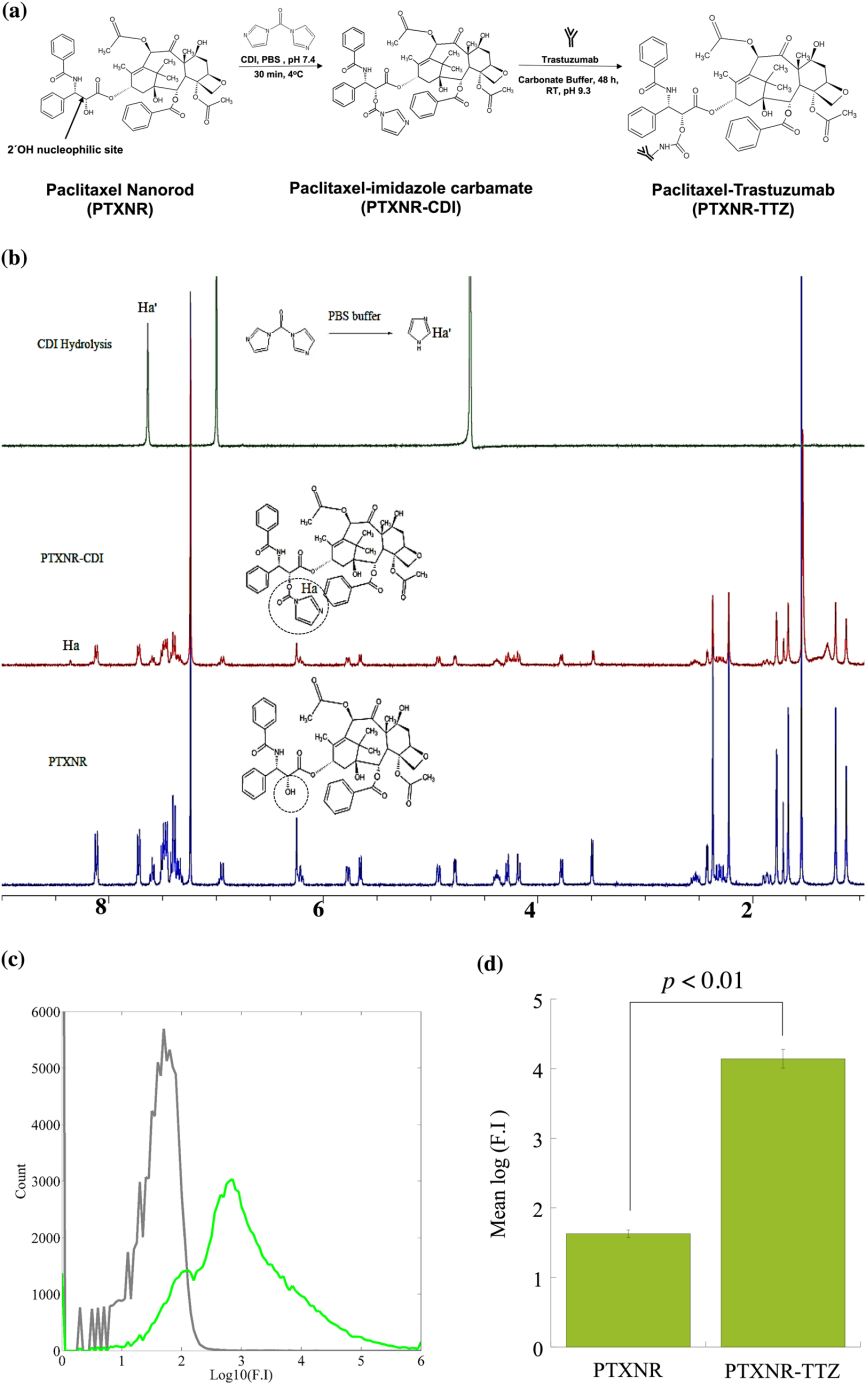
^1^H-NMR analysis to characterize PTX-imidazole carbamate formation: (**a**) Schematic diagram of reactions involved for PTXNR functionalization and TTZ conjugation on the surface of PTXNRs. (**b**) ^1^H-NMR spectra of hydrolyzed carbonyldiimidazole (CDI), PTX-imidazole carbamate (PTXNR-CDI), and PTXNR. The NMR spectra were acquired at room temperature. For characterization of PTXNR and PTXNR-CDI deuterated chloroform (CDCl_3_, δ 7.24) and hydrolyzed CDI deuterated oxide (D_2_O, δ 4.65) were used as reference carrier solvents. (**c**) The fluorescence intensity of PTXNR-TTZ confirms the conjugation of TTZ on the surface of PTXNRs. The F.I. of bare PTXNRs (grey) and Alexa 594 fluorophore bound TTZ conjugated PTXNR (PTXNR-TTZ) (green) shows a significant increase in emission spectra. (**d**) The quantitative mean F.I. of PTXNR-TTZ increases by ~ 14,000 fold (2.54 logarithmic fold) compared to bare PTXNRs indicating TTZ is effectively conjugated on the surface of PTXNRs using a CDI linker.

**Figure 3:**
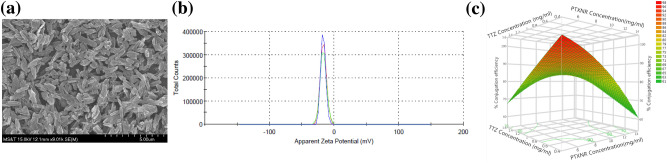
Characterization of PTXNR-TTZ ADNs: (**a**) SEM image of PTXNR-TTZ ADNs. (**b**) The zeta potential (ξ) of PTXNR-TTZ was observed − 17.1 ± 3.83 mV in DI water. (**c**) Contour plot of response surface analysis showing % conjugation efficiency of TTZ on the surface of PTXNRs. In response surface analysis, two factors, PTXNR concentration and TTZ concentration were optimized for the highest conjugation efficiency. Each concentration factor had three levels. For PTXNR, the concentration levels were 5, 10, and 15 mg/ml, and for TTZ the levels were 0.5, 1.0, and 1.5 mg/ml, respectively. Each concentration was replicated at least three times resulting in a total of 27 experimental units. A maximum of ~ 95% conjugation efficiency was obtained using 5 mg/ml PTXNR and 0.5 mg/ml TTZ. The experiment was designed according to a complete randomized (CR) model using JMP statistical software.

### Fluorescence data analysis to confirm the conjugation of TTZ with PTXNR

To confirm the successful conjugation of TTZ with PTXNR, TTZ was tagged with Alexa 594 red fluorescent dye molecule (Invitrogen) according to the manufacturer’s protocol before conjugating with PTXNR. The fluorescence data of both unconjugated bare PTXNR and conjugated Alexa 594 tagged PTXNR-TTZ particles were obtained using a flow cytometer (BD Accuri C6 plus). The fluorescence signal of particles was acquired using a 585/40 bandpass filter with 488 nm laser excitation.

### Optimization of TTZ conjugation using response surface analysis

The optimum conditions for maximum TTZ conjugation efficiency were investigated by the response surface analysis method using JMP statistical modeling software. The design of the experiment involved two factors, initial PTXNR, and initial TTZ concentration. Three levels were assigned to each of the two factors. For initial PTXNR concentration, the levels were 5, 10, and 15 mg/ml, and for initial TTZ concentrations, the levels were 0.5, 1.0, and 1.5 mg/ml, respectively. Each experimental design unit was replicated three times resulting in a total of 27 experimental units. For each experimental unit, a random number was assigned using JMP. The experiments were performed according to a complete randomized design in three days with nine randomly assigned experiments in a single day. Conjugation efficiency of TTZ was chosen to be the yield or response of the experimental design. TTZ conjugation efficiency was calculated using Eq. (1):

1 $${\text{\% }}\;{\text{conjugation}}\;{\text{efficiency}} = \frac{{\left( {{\text{initial}}\;{\text{amount}}\;{\text{of}}\;{\text{TTZ}} } \right) - \left( {{\text{unbound}}\;{\text{amount}}\;{\text{of}}\;{\text{TTZ }}} \right)}}{{\left( {{\text{initial}}\;{\text{amount}}\;{\text{of}}\;{\text{TTZ}}} \right)}} \times100$$

### In vitro therapeutic efficacy

HER2 positive BT-474 and SK-BR-3 breast cancer cells, and HER2 negative MDA-MB-231 breast cancer cells were cultured in hybricare medium (ATCC), McCoys medium (ATCC), and RPMI-1640 (Gibco), respectively supplemented with 10% fetal bovine serum (FBS) and 1% penicillin–streptomycin. MCF-10A normal breast epithelial cells were cultured in mammary epithelial cell basal medium (ATCC) supplemented with mammary epithelial cell growth kit (ATCC). All cells were cultured in a 37 °C and 5% CO_2_ cell culture incubator. Approximately, 10,000 cells per well were plated in 96-well plates and treated with different doses of PTXNR-TTZ, PTXNRs, TTZ alone, PTX solution alone, and PTX solution in combination with TTZ. After 72 h of incubation, cell viability was assessed using MTT (3-(4, 5-dimethylthiazol-2-yl)-2, 5-diphenyltetrazolium bromide, MW 414) assay. MTT reagent was added to each well to convert into an insoluble formazan from water-soluble MTT. After 4 h, sodium dodecyl sulfate (SDS) solution prepared in 0.01 N HCl was added to each well to solubilize the formazan. The percentage of live cells relative to untreated control wells was quantified by measuring the absorbance at 570 nm (BioTek Synergy 2). Cell viability was calculated as a means of six wells containing BT-474 and MDA-MB-231 cells by subtracting the mean background level of wells containing medium only. Nonspecific formation of formazan due to the presence of medium was determined from triplicate wells. The number of viable cells was calculated as follows [Eq. (2)]:

2 $$\% \;{\text{cell viability}} = \frac{{A_{\rm 570\;of\;sample} - A_{\rm 570\;of\;medium } }}{{A_{\rm 570\;of\;live\;cells} - A_{\rm 570\;of\;medium } }} \times 100$$

### In vitro nuclear condensation assay

To further understand cell cytotoxicity of PTXNR-TTZ on breast cancer cells, Hoechst 33342 dye (361/486 nm ex/em) was used to stain the DNA of BT-474, SK-BR-3, and MDA-MB-231 breast cancer cell lines. Approximately, 20,000 cells were seeded in 8 well plates (Corning) followed by 10,000 nM of PTXNR-TTZ treatments for 72 h and incubation with Hoechst (5 µM). Live cells were imaged using a fluorescence microscope (Zeiss Apotome 2.0) equipped with a (450 ± 40) nm wavelength filter cube. The fluorescence images were captured to identify changes in cell nuclei by PTXNR-TTZ treatments.

### Quantitative analysis of synergistic effects of PTXNR-TTZ

We investigated the quantitative effects of PTXNR-TTZ using combination index analysis (CI) by the Chou-Talalay method^55,56^. The CI of PTXNR alone, TTZ alone, and conjugated PTXNR-TTZ were calculated using Eq. (3) and plotted as CI *versus* the fraction of cells being affected.

3 $$CI = \frac{{\left( { Dosage\;of\;PTXNR\;in\;PTXNR - TTZ} \right)}}{{\left( {Dosage\;of\;PTXNR} \right)}} + \frac{{\left( {Dosage\;of\;TTZ\;in\;PTXNR - TTZ} \right)}}{{\left( {Dosage\;of\;TTZ} \right)}}$$

### Apoptosis quantification

The percentage of apoptosis and necrosis in BT-474, SK-BR-3, and MDA-MB-231 cell lines were quantified using the annexin V-FITC and propidium iodide (PI) detection kit. The assay was carried out according to the manufacturer’s instructions. Briefly, cells were seeded into 24-well culture plates (50,000 cells/well), incubated overnight at 37 °C under 5% CO_2_, and treated with PTXNR-TTX (10,000 nM) or culture medium (negative control) for 0, 24, 48 and 72 h. Next, cells were washed using PBS, suspended in 1× binding buffer, and incubated in the dark for 30 min with annexin V-FITC (1:20 dilution) and PI (1:40 dilution). The results of fluorescence-activated cell sorting (FACS) analysis, carried out in a flow cytometer (BD Accuri C6 plus), were analyzed using the BD Accuri C6 plus software. At least three independent tests were performed. The percentage of cell death was calculated from the fluorescence intensity (F.I.) of annexin V-PI. At least 12,000 cells from the gate were acquired and analyzed using the histogram. At least three independent experiments were performed.

### Cell cycle analysis

The effect of the PTXNR-TTZ on the cell cycle of BT-474 cells was investigated after 24, 48, and 72 h of treatments. BT-474 cells without any treatments, PTXNR alone treatment, and TTZ alone treatment were used as controls. The cell cycle analysis protocol was developed by modifying the previously reported method^57,58^. For each sample of the analysis, $2 \times 10^{6}$ cells were seeded in a T25 cell culture flask. After 12 h, PTXNR-TTZ, PTXNR alone, and TTZ alone were added and incubated for 24, 48, and 72 h. The untreated sample was incubated with 1 ml of PBS solution. Before harvesting the cells, 100 µl of 5′-Bromo-2′-deoxyuridine (BrdU) (Alfa Aesar) stock solution per 5 ml of media was added to an active concentration of 40 µM and incubated for 1 h at 37 °C. The culture media was removed completely, and the cells were collected in 15 ml centrifuge tubes. The cells were centrifuged, and the cell pellets were fixed by adding 1 ml of 100% ice-cold ethanol. The cells were stored at 4 °C at least for 30 min. Ethanol was removed after centrifugation at 500× *g* for 5 min and was resuspended in 1 ml of 0.5% Triton X-100 prepared in 2 N HCl. After 30 min of incubation at room temperature, cells were pelleted down to mix with 1 ml of 0.1 M Na_2_B_4_O_7_.10 H_2_O (pH 8.5) solution and was incubated for at least 30 min at room temperature. The cells were pelleted down again and washed with PBS containing 1 mg/ml Bovine Serum Albumin (BSA) and 0.1% Tween-20 (PBST) solution. After washing, cells were resuspended in 50 µl prediluted mouse anti-BrdU monoclonal antibody (Novus Biologicals, Cat. NBP248373) stock solution. After 30 min of incubation in dark, 0.5 ml PBST stock solution was added and washed. A 50 µl prediluted FITC conjugated rabbit anti-mouse secondary antibody (SouthernBiotech, Cat. 617002) stock solution was added to cell pellets and were incubated for 30 min at room temperature in dark. After washing again with PBST solution, cells were resuspended in 100 µl of 1 mg/ml RNAse A (Alfa Aesar) stock solution and incubated for at least 20 min. Finally, 300–500 µl of 35 µg/ml propidium iodide (PI) (Alfa Aesar) was added to cell pellets and incubated in the dark at 37 °C for 45 min. Before analyzing the cells in a flow cytometer (BD accuri C6 Plus), cells were passed through a 40-micron cell strainer (Thermo Scientific) to remove cell aggregates. For flow cytometry analysis, cells were gated to sort out the singlet cells, and only the singlets were gated for subsequent analysis. For acquiring the fluorescent data, 530/30 and 585/40 bandpass filters were used to acquire the BrdU positive and PI-positive cells, respectively using 488 nm of laser excitation.

### Western blot analysis

BT-474 and MDA-MB-231 cells were treated with PTXNR-TTZ, PTXNR alone, and TTZ alone for 48 h at 10,000 nM each for Western blot analysis. The cells were harvested and lysed at 4 °C using 50 mM Tris–HCl, pH 8.0, 150 mM NaCl, and 0.1% Triton X-100, 0.5% sodium deoxycholate and 0.1% sodium dodecyl sulfate (SDS). The lysate protein was quantified using the BCA assay and the BSA calibration curve (SI Fig. 4). For each experiment, 40 µg of each sample was taken and added to an equal volume of 2X Laemmli sample buffer. The cell lysate in the sample buffer was heated at 86 °C for 5 min before running the SDS-PAGE electrophoresis process. Novex Tris–Glycine SDS gels (8–16% and 16%) (Invitrogen) were used for gel electrophoresis. The electrophoresis was performed for 45 min to 1 h at 200 V. The separated proteins were transferred from gel to nitrocellulose membrane using a Power Blotter (ThermoFisher Scientific) according to the manufacturer’s protocol. After the transfer process, the membrane was blocked for 5 h with moderate shaking using 3% BSA protein in TBST buffer [20 mM Tris (pH 7.5), 150 mM NaCl, and 0.1% Tween 20]. The membrane was incubated with a primary monoclonal antibody overnight at 4 °C. GAPDH (ThermoFisher, Cat. MA5-15738, 1: 5000) monoclonal antibody was used as a loading control. Actin (ThermoFisher, Cat. MA5-11869, 1: 2000), caspase -9 (ThermoFisher, Cat PA5-16358, 1:500), caspase-3 (ThermoFisher, Cat. 43–7800, 1:500), cleaved caspase-3 (ThermoFisher Cat. PA5- 23921, 1:1000), XIAP (ThermoFisher, Cat. PA1-84846, 1:1000) and cytochrome-C (ThermoFisher, Cat. MA5-11674, 1:400) primary monoclonal antibodies were used to observe the corresponding proteins of interest. After primary antibody incubation, the membrane was washed 3–5 times for 5 min each with TBST buffer. The membrane was incubated in horseradish peroxidase (HRP) enzyme-conjugated secondary anti-mouse (ThermoFisher Cat.A27025, 1:10000 or anti-rabbit monoclonal antibody (Cell Signaling Technology, Cat.7074P2, 1:10000) depending on the primary antibody host species for 1 h followed by washing with TBST washing buffer. The electro-chemiluminescent substrate (Super Signal West Dura, ThermoFisher, and Cat. 34075) was added to the membrane and incubated for 5 min. The electro-chemiluminescent signal was captured using a Bio-Rad gel imager. The signals were analyzed by image processing using Fiji (Image J) software.

**Figure 4:**
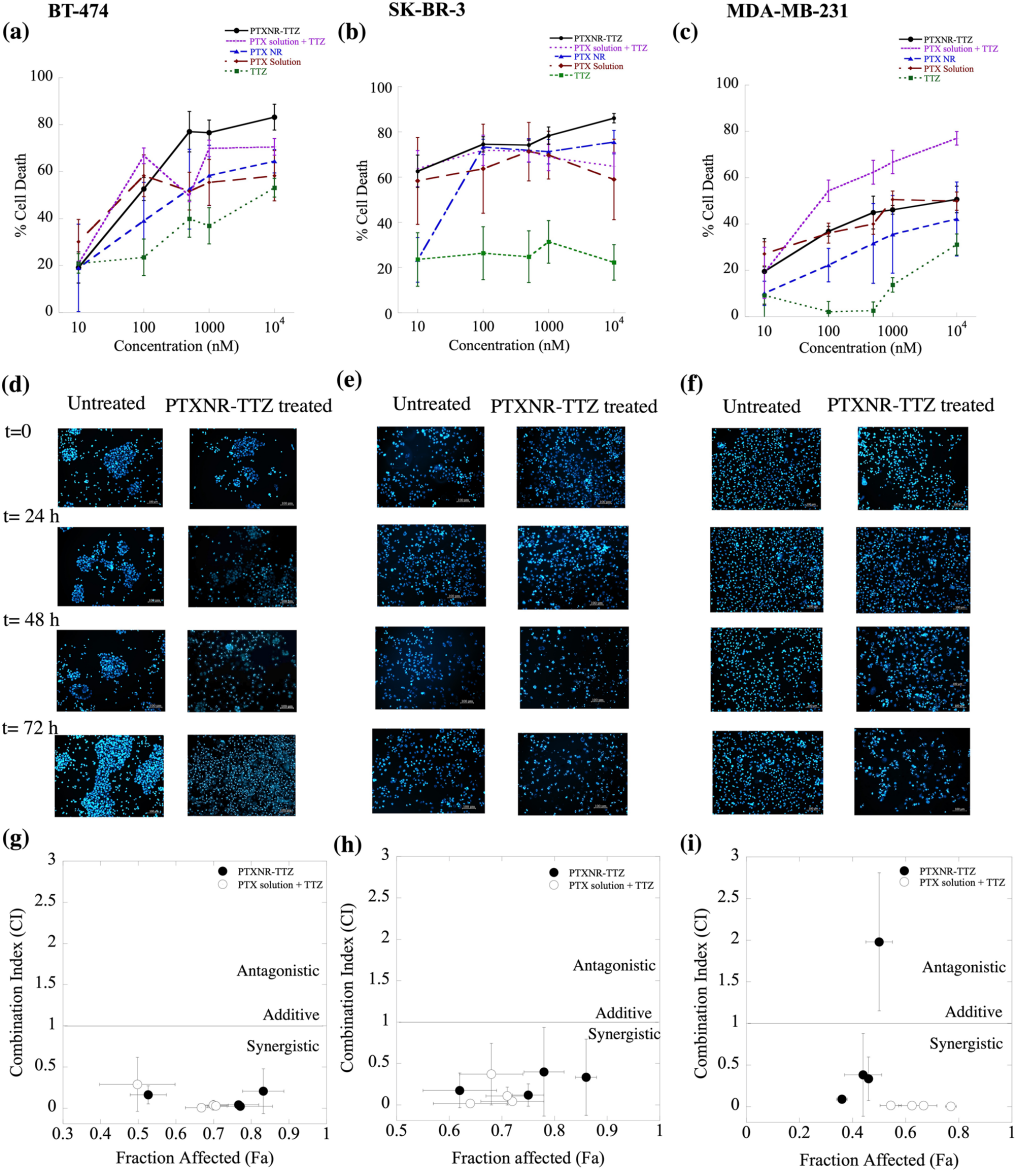
(**a**)**–**(**c**) In vitro anti-cancer efficiency of PTXNR-TTZ ADNs: The cytotoxic effects of PTXNR-TTZ (closed circle), PTXNR (triangle), PTX solution (diamond), and TTZ (square) on the growth of (**a**) BT-474, (**b**) SK-BR-3, and (**c**) MDA-MB-231 cells. Each experiment was replicated at least n = 6 times, and the average data is presented with mean ± standard deviation. The *p* values are given in SI Table 3. (**d**)**–**(**f**) The nuclear morphology of apoptotic cells stained with Hoechst 33342: Cells transfected with PTXNR-TTZ were stained with Hoechst 33342 72 h post-transfection and observed under a fluorescence microscope. The combined treatment with PTXNR-TTZ displayed the increased amount of nuclear fragmentation in (**d**) BT-474, (**e**) SK-BR-3, and (**f**) MDA-MB-231 cells. Untreated control cells remained uniformly stained with round and undamaged nuclei. The figures represent one of three independent experiments. (**g**)**–**(**i**) Combination index (C.I.) analysis of PTXNR-TTZ ADNs: The C.I. plot as a function of each fraction of cells affected by PTXNR-TTZ particles in (**g**) BT-474, (**h**) SK-BR-3, and (**i**) MDA-MB-231 cells. A C.I. value = 1; additive effect, < 1; synergistic effect, > 1; antagonistic effect.

### Statistical analysis

Data were analyzed using JMP statistical software (version 15) and presented as mean ± standard deviation. To observe the statistically significant differences in PTXNR-TTZ treated cells compared to controls, we performed the analysis of variance (ANOVA) using a one-tailed Student’s t-test with a *p* value ≤ 0.05 and a confidence interval ≤ 95%. The TTZ conjugation efficiency analysis was performed using a complete randomized (CR) model. The final conjugation efficiency data was predicted using a response surface analysis using JMP statistical software.

## Results

### Synthesis of PTXNRs

The SEM images of PTXNRs as shown in Fig. 1a confirms the elongated rod shape PTX drug NRs. The average diameter and length of PTXNRs were (96.87 ± 33.08) and (503.42 ± 210) nm, respectively (Fig. 1b). The size distribution plots (Fig. 1c, d) confirm the moderately narrow dispersity of the particle diameter and length. The polydispersity index (pDI) of the particles was measured as 0.20 ± 0.02. PTXNRs showed high colloidal stability in DI water having a zeta potential value of (− 32.60 ± 4.82) mV (Fig. 1e, SI Table 1). In 0.15 M PBS solution, the zeta potential value was measured (− 13.40 ± 2.81) mV, indicating the stability of nanoparticles even in high salt concentrations (SI Table 1).

### Confirmation of linker conjugation with PTXNRs using ^1^H NMR analysis

We activated the 2′ OH site of PTXNRs to form an imidazole carbamate intermediate for subsequent conjugation step with the ε- amino group in the lysine side chain of TTZ (Fig. 2a). In ^1^H NMR analysis, imidazole carbamate intermediate linkage was confirmed by the peak of the imidazole ring proton in ‘a’ position (Ha) at 8.35 ppm (Fig. 2b)^59,60^. In a parallel side reaction, CDI hydrolyzes in the aqueous phase of conjugation reaction with TTZ. Hydrolyzation of CDI produces a free imidazole ring and might interfere in the NMR analysis as a free surface-bound residuals. To further confirm the linkage of the imidazole carbamate with PTXNRs, we performed the NMR analysis of hydrolyzed CDI residuals. Residual imidazole group was identified by the peak of the imidazole proton in “a′” position (Ha′) at 7.6 ppm^59^. So, the shift of NMR peak at 8.35 ppm represents only the Ha proton, and hence, confirms the successful linkage of imidazole carbamate at 2′ OH site of PTXNR particles forming the PTX-imidazole carbamate intermediate (PTXNR-CDI) particles.

### Confirmation of TTZ conjugation with PTXNR-CDI

The ε-amino group in lysine residue of TTZ acts as an active electrophile at the reaction pH and attacks the CDI activated site of PTXNR through nucleophilic substitution reaction. Attack by the electrophile amine group releases the imidazole ring but leaves the carbonyl group resulting in a one-carbon spacer forming a stable carbamate linkage. To confirm the TTZ conjugation, Alexa 594 fluorescent molecule was conjugated with TTZ. The bare unconjugated PTXNR showed a mean autofluorescence intensity of 42.60 a.u. (arbitrary unit). For Alexa 594 fluorophore bound PTXNR-TTZ, the mean fluorescence intensity was measured 13,889.46 a.u. (Fig. 2c) that is 326 fold higher than the mean fluorescence intensity of bare PTXNR confirming a successful conjugation of TTZ with PTXNRs (Fig. 2d). PTXNR-TTZ showed good dispersibility in both water and PBS (SI Fig. 5).

**Figure 5:**
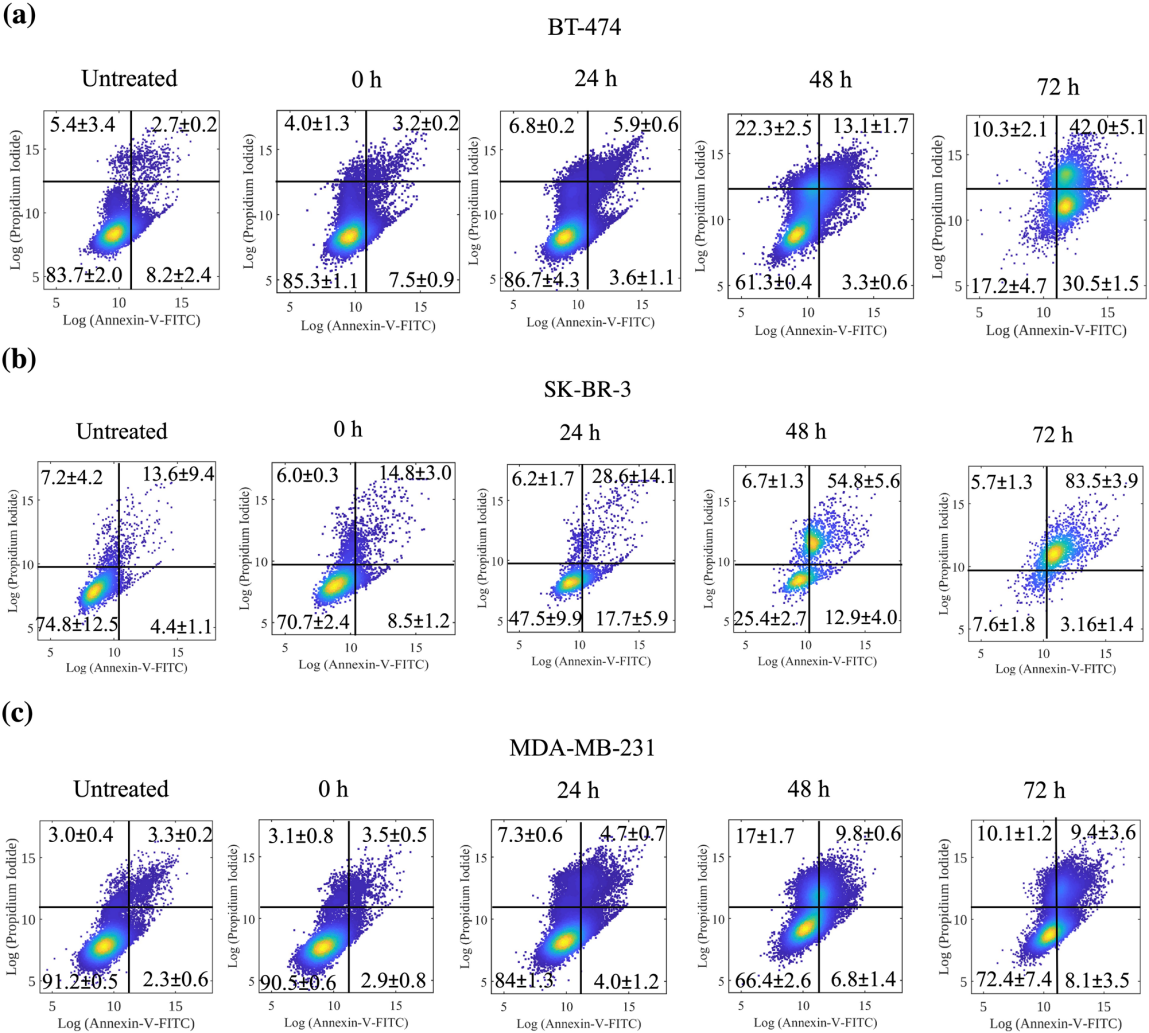
Evaluation of apoptosis induction. Annexin V-FITC analysis of (**a**) BT-474, (**b**) SK-BR-3, and **(c)** MDA-MB-231 cells untreated and treated with PTXNR-TTZ for 0, 24, 48, and 72 h. Results represent one of three independent experiments. Viable cells are represented in the lower left quadrant. Early apoptosis (Annexin + / PI −) is shown in the lower right quadrant for each panel, while late apoptosis (Annexin + / PI +) is shown in the upper right quadrant. Necrosis (Annexin − / PI +) is shown in the upper left quadrant. Values are presented as means ± SD of triplicate experiments. Detailed statistical analysis (*p* < 0.05) of the data are shown in SI Table 4.

### Optimization of TTZ conjugation efficiency

The SEM image of the PTXNR-TTZ particles confirms the consistency in the size and shape of particles after lyophilization (Fig. 3a). The zeta potential of the PTXNR-TTZ in DI water was measured − 17.1 ± 3.83 mV (Fig. 3b, SI Table 1). In PBS, the zeta potential was measured − 9.5 ± 0.02 mV (SI Table 1). The conjugated particles had a PDI of 0.21 ± 0.11, showing significant homogeneity in size and shape. Optimization of TTZ conjugation efficiency was performed using response surface analysis (Fig. 3c). The surface plot shows that the vicinity of lower concentrations of both initial PTXNR and TTZ result in 90–95% of conjugation efficiency, whereas the higher initial concentrations predict lower conjugation efficiency. The analysis suggests that the higher concentration of PTX results in precipitation of nanoparticles and less available surface for conjugation and several vacant reactive sites on the nanoparticle surface, whereas the higher concentration of TTZ produces excess unbound TTZ in the reaction medium. The maximum conjugation efficiency of TTZ to PTXNR was calculated as 95.24 ± 1.40% (SI Table 2), and hence, considered the optimum amount to use for subsequent experiments. The optimum condition for the maximum conjugation efficiency was 5 mg/ml and 0.5 mg/ml initial concentrations of PTXNR and TTZ, respectively. The drug to antibody ratio (DAR) of the conjugated particle was 4.0 ± 0.53 on a weight basis (molar ratio = 681.67 ± 90.23) (SI Table 2).

### In vitro anticancer efficacy

To determine the anti-cancer efficacy in vitro, the dose–response cytotoxicity was measured using PTXNR-TTZ, PTX NR alone, TTZ alone, PTX solution alone, and co-treatments of PTX and TTZ solutions in BT-474, SK-BR-3, and MDA-MB-231 cells (Fig. 4). The treatment of BT-474, MDA-MB-231, SK-BR-3, and MCF-10A with PTXNR-TTZ resulted in a concentration-dependent effect (Fig. 4). The cells were treated with PTXNR-TTZ and the following controls: cotreatment with PTX and TTZ solutions, PTX NR alone, PTX solution alone, and TTZ solution alone. The treatments using PTXNR-TTZ and controls showed dose-dependent response curves. The % cell death quantified using MTT assay is shown by the mean ± standard deviation. Differences between PTXNR-TTZ treatment and controls were analyzed using one way ANOVA followed by Student’s t-test method. *** projects significant differences with *p* < 0.05 and 95% confidence interval (SI Table 3). PTXNR-TTZ did not show significant differences in BT-474 and SK-BR-3 cells at concentrations ≤ 1000 nM compared with controls. A significant difference (*p* < 0.05) in the % of cell death is found at 10,000 nM of PTXNR-TTZ inhibiting > 83% of BT-474 cells (Fig. 4a). The cytotoxicity of PTX solution and TTZ was dose-dependent with the mean cell death of ~ 70% compared to that of PTXNR-TTZ. The mean cell death was increased from ~ 30% to 70% when the cells were treated with PTX NR or PTX solution alone. PTX solution and the co-treatment of PTX solution with TTZ showed slightly (~ 10%) higher mean cell death at concentrations ≤ 200 nM than that by the treatment using PTXNR-TTZ in BT-474 cells. PTXNR-TTZ may maintain a slow and sustained release of drugs owing to the dissolution from the nanorod conjugates. In our previous study, we showed similar in vitro drug release mechanisms of camptothecin from CPT-PCL-TTZ NRs following Fick’s diffusion model^52^. The low bioavailability of PTXNR-TTZ at concentrations ≤ 200 nM might cause lower cell death than PTX solution and the combination of PTX and TTZ solution. TTZ solution caused ~15-50% cell death .

TTZ alone exhibited very low toxicity in SK-BR-3 cells with cell death observed to be over 20% following exposure up to 10,000 nM concentration of the drug (Fig. 4b). The cytotoxic effect of PTNR-TTZ was more evident in SK-BR-3 than BT-474 cells, due to selective binding of the conjugate to cells that overexpress HER-2. These results are in good agreement with previous studies that showed that when maytansinoid was conjugated with TTZ (T-DM1), binding with HER-2 receptors was more specific both in vitro and in vivo than the free monoclonal antibody^61^. Other studies have confirmed the selective antiproliferative activity of PTX conjugated with TTZ in micelle drug carriers toward HER-2-positive SK-BR-3 cells compared to MDA-MB-231 cells^62^.

The addition of PTXNR-TTZ, PTX NR alone, PTX solution alone, and TTZ solution caused 20-50% cell death in HER2- MDA-MB-231 cells in the dose range of 1–10,000 nM (Fig. 4c). The mean cell death using PTXNR-TTZ did not differ significantly compared to single-agent treatments. Increasing cytotoxicity was observed by the treatment of PTX and TTZ solutions in a dose-dependent fashion. These data indicate non-specific therapeutic efficiency in HER2 negative MDA-MB-231 cells. The percentages of cell death in MCF-10A control cells varied between 5–40% at concentrations between 0.1–10,000 nM (SI Fig. 6) showing moderate toxicity in non-cancerous cells. A similar level of toxicities has been reported using paclitaxel^63^, taxane^64^, and nanoparticles^65^ in MCF-10A cells in vitro.

**Figure 6:**
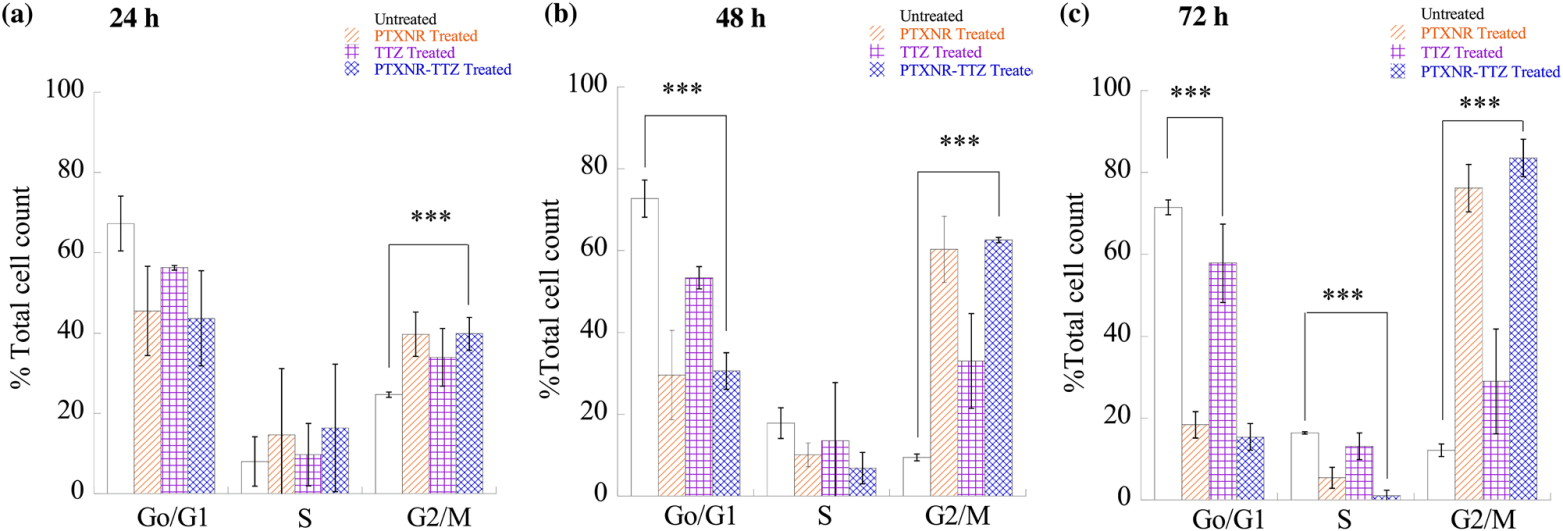
Cell cycle analysis: BT-474 cells were treated with PTXNR-TTZ, PTXNR alone, and TTZ alone. Cell cycle analysis was performed by flow cytometry after (**a**) 24 h, (**b**) 48 h, and (**c**) 72 h of treatment. Each experiment was replicated at least n = 2 times, and the average cell count data are presented as mean ± standard deviation. Statistically significant differences are shown using *** (*p* < 0.05; detailed *p* value calculations are shown in SI Table 5).

The half-maximal inhibitory concentrations (IC_50_), as defined as the concentration needed to kill 50% of cells, were found 34, 106, 609, and 9000 nM for co-treatment using PTX and TTZ, PTXNR-TTZ, PTX NR alone, and TTZ alone, respectively in BT-474 cells indicating a synergistic therapeutic efficiency using ADNs compared to the individual treatments (Fig. 4a). The IC_50_ values of PTX and TTZ solutions, PTXNR-TTZ, and PTX NR alone were 54, 31, and 51 nM, respectively in SK-BR-3 cells (Fig. 4b). The IC_50_s of PTX and TTZ co-treatments was slower than PTXNR-TTZ, which might be the consequence of slow drug release of PTX from PTXNR-TTZ due to its hydrophobicity. The IC_50_ of PTXNR-TTZ is higher in BT-474 than SK-BR-3 most likely due to almost tenfold HER2 higher concentrations in the cell membrane of BT-474 than that in SK-BR-3 cells^66^. MDA-MB-231 cells were resistant to PTXNR-TTZ exhibiting an IC_50_ > 3000 nM indicating intrinsic TTZ resistance in vitro (Fig. 4c). PTXNR alone and TTZ solution alone inhibited roughly 40% and 30% of the MDA-MB-231 cell growth, respectively. The cytotoxic effects of PTX solution were similar in both cell lines showing a dose-dependent cytotoxicity from 30 to 50%. PTXNR effectively killed more BT474 cells from 20 to 60% in dose-dependent manner than that in MDA-MB-231 cells from 10 to 30%. All these results suggest that PTXNR-TTZ exerts specific cytotoxicity in HER2 positive breast cancer cells.

### Microscopic analyses on cell cytotoxicity

Chromatin condensation analysis by Hoechst staining was performed to study the cytotoxic effect of PTXNR-TTZ on BT-474 (Fig. 4d), SK-BR-3 (Fig. 4e), and MDA-MB-231 cells (Fig. 4f). During apoptosis, the chromatin becomes inert, highly condensed, undergoes fragmentation, and gets packaged into apoptotic bodies. The morphological changes induced by apoptosis can be detected by the blue-fluorescent Hoechst 33342 dye, which brightly stains the highly condensed, dense chromatin of apoptotic cells in comparison to the chromatin of untreated cells. After treatment with PTXNR-TTZ (10,000 nM) for 72 h, the number of condensed nuclei increased with time compared to untreated control cells exhibiting uncompromised cell nuclei after 72 h. The results indicate that PTXNR-TTZ is effective in inducing apoptosis in breast cancer cells.

### The effects of PTXNR-TTZ is synergistic in HER2 positive breast cancer cells

The combination effects of PTXNR-TTZ and the cotreatments using PTX and TTZ solutions were evaluated in BT-474 (Fig. 4g), SK-BR-3 (Fig. 4h), and MDA-MB-231 cells (Fig. 4i) using the combination index (CI) analysis. The effects of the drugs were analyzed to determine whether a synergistic effect occurred by the treatment of PTXNR-TTZ in HER2 positive breast cancer cell lines. On the plot, any point showing CI < 1 indicates a synergistic effect, and the values showing CI > 1 are considered antagonistic. PTXNR-TTZ treatment in BT-474 cells resulted in CI values ranging from 0.16 ± 0.1 at 100 nM to 0.02 ± 0.02, 0.04, ± 0.04, and 0.2 ± 0.3 at 500, 1000, and 10,000 nM, respectively, indicating synergistic effects of drugs in the concentration range being tested (Fig. 4g). SK-BR-3 cells had relatively higher CI values than BT-474 with CI ranging from 0.12 ± 0.14, 0.60, ± 0.68, and 0.4 ± 0.6, and 0.34 ± 0.46 at 100, 500, 1000, and 10,000 nM, respectively (Fig. 4 h), indicating reduced synergy against breast cancer cells with lower HER2 overexpression on the cell membrane than BT-474 cells^66^. In contrast, very different CI *vs.* fraction affected line was observed in MDA-MB-231 cells showing increased CI values with increasing fraction affected cells up to 0.8 beyond which antagonistic effects were observed indicating a combination of PTX NR and TTZ is irrelevant in this cell line (Fig. 4 i). PTXNR-TTZ resulted in an antagonistic interaction (CI = 2 ± 0.8) at 10,000 nM in MDA-MB-231 cells, representing antagonism at high concentrations. Co-treatments of PTX with TTZ solutions showed synergistic antitumor effects in all three cell lines suggesting that the drug combination was effective for anticancer therapies.

### Measurement of apoptotic populations using Annexin V-FITC and PI

Annexin V and PI double-stained cells collected from the flow cytometry assay were observed for distinguishing live, early apoptosis, late apoptosis, and dead cell subtypes at the individual incubation time points of 24, 48, and 72 h. The Annexin V assay revealed that PTXNR-TTZ induced early and late stages of apoptosis in BT-474 cells (Fig. 5a). The cell distributions in live, early apoptosis, late apoptosis and dead cells are 83.7 ± 2.0%, 8.2 ± 2.4%, 2.7 ± 0.2%, and 5.4 ± 3.4%, respectively in untreated BT-474 cells. Induction of apoptosis by PTXNR-TTZ in BT-474 cells in a time-dependent manner was observed. The early apoptosis (Annexin V-FITC) plots show the BT-474 cell distribution of 7.5 ± 0.9%, 3.6 ± 1.1%, 3.3 ± 0.6%, and 30.5 ± 1.5% at t = 0, 24, 48, and 72 h, respectively following PTXNR-TTZ treatments. An increase in late apoptosis and dead cells was seen in all treated cells in comparison to the untreated cells. After 24 h of treatment, the percentage of late apoptotic cells increased from 3.2 ± 0.2% to 5.9 ± 0.6%. As the treatment duration reached 48 h, the percentage of apoptotic cells was increased further to 13.7 ± 1.7% and increased even more, to 42.0 ± 5.1%**,** after 72 h. The statistical differences are provided in SI Table 4. In untreated SK-BR-3 cells, the cell distribution in live, early apoptosis, late apoptosis, and dead cells are 74.8 ± 12.5%, 4.4 ± 1.1%, 13.6 ± 9.4%, and 7.2 ± 4.2%, respectively indicating a very low number of early, late apoptotic, and dead cells (Fig. 5b). However, the apoptotic cell distribution was increased after treatment with PTXNR-TTZ. The percentage of late apoptotic cells increased gradually with increasing treatment duration, being 28.6 ± 14.1%, 54.8 ± 5.6%, and 83.5 ± 3.9%, following 24, 48, and 72 h of incubation, respectively. The increase in cell distribution indicated a time-dependent increase in late apoptotic cells, parallel to that noted in BT-474 cells. Untreated MDA-MB-231 cells showed a distribution of 91.2 ± 0.5%, 2.3 ± 0.6%, 3.3 ± 0.2%, and 3.0 ± 0.4% in live, early apoptosis, late apoptosis, and dead cells, respectively (Fig. 5c). The percentage of late apoptosis increased to 3.5 ± 0.5%, 4.7 ± 0.7%, 9.8 ± 0.6%, and 9.4 ± 3.6% for 0, 24, 48 and 72 h of PTXNR-TTZ treatment, respectively. The % of apoptotic MDA-MB-231 cells displayed only a slight increase in cell distribution as a result of treatment with PTXNR-TTZ. Thus, the overall cell population shift indicated that PTXNR-TTZ caused a significant increase in apoptosis in HER2 positive breast cancer cells.

### PTXNR-TTZ arrests HER2 positive breast cancer cells in the G2/M phase

We quantified the population of BT-474 cells arrested in G0/G1, S, and G2/M cell cycle phases after treatment with PTXNR-TTZ, PTXNR alone, and TTZ alone (Fig. 6, SI Figs. 7–9 and SI Table 5). PTXNR-TTZ arrested 39.90, 62.56, and 83.55% of BT-474 cells in the G2/M phase after 24, 48, and 72 h treatments, respectively. Conversely, it decreased the proportion of the G0/G1 phase from 43.70 to 30.6 to 15.39% at 24, 48, and 72 h, respectively. PTXNR alone caused 39.76, 60.31, 76.18% arrest in the G2/M phase after 24, 48, and 72 h, respectively. TTZ alone treatment arrested 56.25, 53.39, 57.83% of BT-474 cells in the G1 phase after 24, 48, and 72 h, respectively. The cell cycle data confirms that PTXNR modulates the mitotic cell cycle arrest, or cell growth inhibition, and eventually apoptosis in BT-474 breast cancer cells.

### PTXNR-TTZ downregulates anti-apoptotic protein and induces apoptosis in a caspase-dependent intrinsic pathway

**Figure 7:**
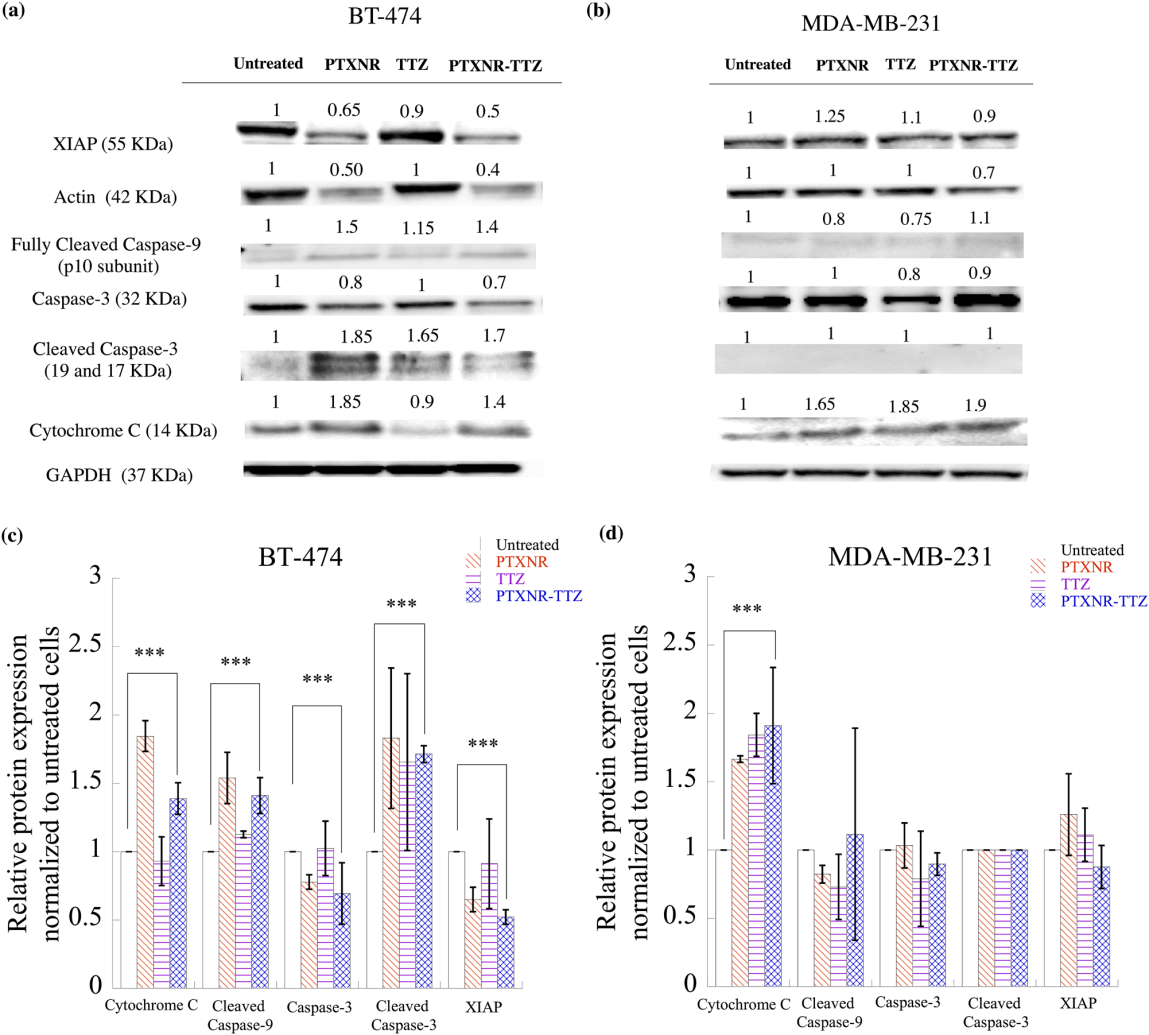
Western blot analysis: The cell signaling protein expressions of (**a**) BT-474 and (**b**) MDA-MB-231 cells using PBS, PTX alone, TTZ alone, and PTXNR-TTX treatments. The relative intensity of XIAP, actin, cleaved caspase-9, caspase 3, cleaved caspase 3, and cytochrome C is analyzed after 48 h of treatment in (**c**) BT-474 and (**d**) MDA-MB-231 cells using ImageJ/ Fiji. For each protein expression the experiment was replicated at least n = 2–4 times and the average data is presented as mean ± standard deviation. The relative protein expressions are normalized to the untreated control cells and to the housekeeping GAPDH protein expression (hypothesized expression mean, µ = 1). The *p* values for overexpression or downregulation of cytochrome-C, cleaved caspase-3, and XIAP in BT-474 are 0.039, 0.003, and 0.000049, respectively. The *p* value for the overexpression of cytochrome-C in MDA-MB-231 is 0.047. ***Represents *p* < 0.05 (for detailed *p* value calculations, please see SI Table 6).

We found that > 80% of BT-474 cells were inhibited and arrested in the G2/M phase by PTXNR-TTZ ADN treatments. We investigated if the prolonged cell cycle arrest in the G2/M phase induced programmed cell death or apoptosis in cancer cells and the mechanisms of apoptotic induction by PTXNR-TTZ ADNs. PTXNR-TTZ induced apoptosis in HER2 positive BT-474 cells in an intrinsic cytochrome-C mediated pathway (Fig. 7a, c and SI Fig. 10a). In BT-474 cells, after 48 h of treatment using PTXNR-TTZ and PTX NR, we observed a significant release of cytochrome-C than that of untreated cells. We further observed a significantly higher expression of initiator apoptotic protein fully cleaved caspase-9. The lower expression of procaspase-3 was correlated with the upregulation of cleaved or active form of caspase-3. The observation is in agreement with the time-dependent expression study of caspase-3 on taxane treated HER2 positive SK-BR-3 cell line^67^. In BT-474 cells, the anti-apoptotic protein XIAP was downregulated by PTXNR-TTZ and PTXNR treatments in comparison to untreated and TTZ treated control cells. XIAP is a natural inhibitor of caspase-3 and caspase-9^47,68–70^. High XIAP activity may cause the inactivation of early apoptotic protein caspase-9 and inactivation of active caspase-3 at the later phase of apoptosis. The significant downregulation of XIAP by PTXNR-TTZ treatment facilitates the late apoptosis by overexpressing the effector caspase-3 apoptotic protein that attributes to the synergistic cytotoxic effect in BT-474 cells. The high concentration of PTXNR and PTXNR-TTZ significantly downregulated the actin production in BT-474 cells compared to MDA-MB-231 cells. In MDA-MB-231 cells, although the cytochrome-C release was observed for both PTXNR-TTZ and PTXNR treatments, there was no significant change in the expression of active caspase-9 suggesting that the apoptosome was not activated in the cytosolic area (Fig. 7b, d and SI Fig. 10b). The procaspase-3 expression in PTXNR-TTZ treated cells was not statistically different from that of untreated cells. Eventually, the cleaved form of the caspase-3 signal was not observed at all with the individual drug treatment and PTXNR-TTZ treatment. XIAP activity was observed in individually treated cells as well as in PTXNR-TTZ treated cells. XIAP activity in the drug-treated cells was found to be as high as that in untreated MDA-MB-231 cancer cells. XIAP is a potent anti-apoptotic protein and abnormal expression of XIAP can block the cell death pathways^47^.

## Discussion

Many chemotherapeutic drugs including PTX is a water-insoluble drug that results in low circulation time, poor bioavailability in the tumor tissue, and higher administrative dosage^71–73^. Nano-sized drug carriers, liposomes, polymeric nanoparticles, and micelles have been designed over the past decades to improve water dispersibility, stability, and blood circulation half-life of chemotherapeutic drugs^74–77^. However, most nanoparticles suffer from poor specificity targeting only 1.6% of the administered dose of the linked small molecule drug to be delivered in the targeted tumor vicinity^78,79^. In this study, we designed ADNs by conjugating TTZ monoclonal antibodies on the surface of PTXNRs through a single carbonyl group spacing with > 95% conjugation efficiency. PTXNR-TTZ was synthesized in two steps. First, PTX NRs were synthesized by phase separation of PTX drug solution from ethanol into water. This method is simple and scalable. Secondly, PTXNRs were reacted with CDI linker in water to form intermediate PTXNR-carbamate followed by coupling with the amine groups of TTZ using carbodiimide chemistry that forms PTXNR-TTZ ADN^80,81^. The reaction is chemically defined, efficient, and scalable with an overall yield of > 80% and a high PTX: TTZ w/w ratio ≈ 4 (molar ratio ≈ 682). The cytotoxic payload in ADNs is 170-fold more than conventional antibody–drug conjugates (molar ratio ≈ 4) which improves the ability to deliver more active drugs at the target site and greater in vitro potency. The PTXNR-TTZ ADN showed greatly enhanced water dispersibility (> 1 mg/ml) compared to PTX aqueous solution, indicating little aggregation in water^52^.

PTXNR-TTZ ADNs are designed to target HER2 receptors overexpressed in breast cancer cells so that the ADN binds to target cells, are internalized via endocytosis, and exerts apoptosis mediated therapeutic effects^10,48,50,51,82–95^. The NRs, when coated with TTZ, exhibit high binding toward HER2-overexpressing breast cancer cells^53,96^. The adhesion strength of NRs has been shown high both experimentally and by theoretical modeling^92,97–100^. After targeting and internalization, PTXNR-TTZ ADNs activated intrinsic apoptosis pathways and inhibited > 80% of HER2 positive BT-474 and SK-BR-3 cells which was 43% more than the cytotoxicity in HER2 negative MDA-MB-231 cells. No difference in cytotoxicity was observed for PTXNR-TTZ, unconjugated PTXNR, PTX solution alone, and TTZ alone treatments in MDA-MB-231 cells, indicating non-specific cell death. The inhibitory effect of PTX and TTZ co-treatments is concentration-dependent inducing non-specific cell death in both HER2 positive and HER2 negative breast cancer cells after 72 h of treatment. The IC_50_ values of PTXNR-TTZ in BT-474 cells and SK-BR-3 cells are lower than single drug treatments which are consistent with the previously published literature. The IC_50_s of PTX vary between 2–200 nM in breast cancer cells due to differential basal level expressions of β-tubulin subunits of microtubules^101–103^, sensitivity of PTX toward binding with β-tubulin^101^, the expression of tau proteins involved in microtubule polymerization^104^, and HER2 expression levels^105^. PTXNR-TTZ inhibits tubulin polymerization and microtubule formation, blocks the cell cycle in G2/M and induces apoptosis.

The CI analysis showed that PXNR-TTZ augments apoptosis in BT-474 cells synergistically indicating an induction in apoptosis through multiple pathways including intrinsic caspase-dependent pathway and cell cycle arrest. PTX and TTZ arrest cells in G2/M and G0/G1 phase, respectively by disrupting the microtubule dynamics and binding with the HER2 extracellular domain^106–110^. Actin polymerization is a universal mechanism to drive cell cycle in dividing cells including the accelerated outgrowth of breast cancer cells^1–3^. PTX, as microtubule-binding drugs, stabilizes microtubules^4,5^, blocks their dynamics^6,7^, and induces apoptosis in cancer cells^8,9^. PTX has been widely used to treat a variety of cancers, including breast cancer, head and neck cancer, gastroesophageal cancer, Kaposi's sarcoma, leukemia, lymphoma, non-small cell lung cancer, and ovarian cancer^10^. Nevertheless, the natural or acquired resistance to PTX limits its therapeutic use. This fact, together with the key role of microtubule dynamics in cancer growth, led to the concept that PTXNR-TTZ directly inhibits actin polymerization causing apoptosis in cancer cells. Using the densitometry of Western blot results, we show that HER2 + breast cancer cells treated with PTXNR-TTZ contained a reduction in actin polymerization by ∼60% compared with untreated cells. In this study, individual treatment using PTXNR alone and TTZ alone arrested 76% and 57% of BT-474 cells in G2/M and G0/G1, respectively after 72 h of treatment. In contrast, PTXNR-TTZ arrested 83.5% of BT-474 cells in G2/M after 72 h of treatment indicating dominant effects of PTX in sensitizing and synchronizing cells in the G2/M phase which correlates with the percentage of apoptotic dead cells after PTXNR-TTZ treatment^111–113^. The cleaved caspase-3 and cytochrome C were significantly increased, while anti-apoptotic XIAP was decreased in BT-474 cells after 72 h of PTXNR and PTXNR-TTZ treatments compared with untreated control. Cytochrome-C binds to apoptosis protease-activating factor-1 (APAF1), inducing its oligomerization to form apoptosome that recruits and activates an apoptosis initiator protein, caspase-9, cleaves caspase-9 into its active small subunits and initiates the late apoptotic caspase cascade reactions^47,70,114,115^. The cleaved caspase-9 eventually activates the late apoptotic protein caspase-3^116–118^. The overexpression of cleaved or active form of caspase-3 ultimately confirms the late phase of apoptosis in BT-474 cells using PTXNR-TTZ treatment^116,119–121^. In the late phase, apoptosis is facilitated by two-fold downregulation of anti-apoptotic XIAP protein after PTXNR-TTZ treatment compared to that in the untreated control. In contrast, MDA-MB-231 cells did not show any sign of mid- or late phase apoptosis although having overexpression of the cytochrome-C protein. No changes in XIAP expression was observed in MDA-MB-231 cells treated with PTXNR-TTZ. XIAP, a key member of the inhibitor of apoptosis protein (IAP) family^122^, has been shown to be a direct inhibitor of caspase-3 and to interfere with the cytochrome-C pathway by inhibiting caspase-9 in lung cancer cells^123–125^, and leukemia cells^126–129^. In this study, the downregulation of XIAP and activation of caspase cascades in BT-474 cells by PTXNR-TTZ has proven to be an effective approach for the treatment of HER2 positive breast cancer cells.

## Conclusion

The first synthesis of PTXNR-TTZ ADNs and its therapeutic efficiency in vitro are presented which induces higher apoptosis responses than single drug treatments in HER2 positive breast cancer cells. The use of PTXNR of ~ 95 nm in diameter and 500 nm in length remove the compromising need for toxic organic carrier solvent for intravenous administration of PTX alone. The particles are stable in the aqueous phase that makes it suitable for intravenous administration without any need for any organic solvent. The surface of PTXNR was functionalized by CDI activation reaction in the aqueous phase and successfully conjugated TTZ without altering the size and shape of the nanoparticles. PTXNR-TTZ synergistically inhibited HER2 positive breast cancer cells. The IC_50_ values of PTXNR-TTZ are 31 and 106 nM in SK-BR-3 and BT-474 cancer cells, respectively which facilitates lower dose, and hence, fewer side effects than individual treatments. PTXNR-TTZ arrested > 83% of the proliferating cells in the G2/M phase of the cell cycle after 72 h of the treatment, which was well corroborated with cell cycle analysis. The higher number of cells (> 80%) arrested in the G2/M phase after PTXNR-TTZ treatment than individual treatments using PTXNR and TTZ (arrests in the G0/G1 phase) confirms the synergistic crosstalk between PTX and TTZ. The Western blot analysis demonstrates that PTXNR-TTZ activates the effector caspase proteins in HER2 positive breast cancer cells, and downregulates the anti-apoptotic XIAP facilitating caspase-dependent apoptosis followed by the cell cycle arrest in the G2/M phase. Taken together, it is envisaged that the results from this study will facilitate the rational design of combination therapy through a novel design of ADNs.

## Supplementary Information

Supplementary Information
