# Supplementary material for: Antibody–drug nanoparticle induces synergistic treatment efficacies in HER2 positive breast cancer cells
Source: Sci Rep. 2021 Apr 1;11:7347. doi: 10.1038/s41598-021-86762-6 (PMC8016985; doi:10.1038/s41598-021-86762-6)
Supplement: Supplementary file 1 — Supplementary Information [file 41598_2021_86762_MOESM1_ESM.pdf]

# Supplementary Information

# Antibody-Drug Nanoparticle Induces Synergistic Treatment Efficacies in HER2 Positive Breast Cancer Cells

Muhammad Raisul Abedin,[a] Kaitlyne Powers,[a] Rachel Aiardo,[a] Dibbya Barua,[b] and Sutapa Barua[a, #]

[a] Department of Chemical and Biochemical Engineering

[b] Department of Electrical and Computer Engineering

Missouri University of Science and Technology

Rolla, MO 65409

[#] To whom correspondence should be addressed. Email: baruas@mst.edu. Department of Chemical & Biochemical Engineering, 110 Bertelsmeyer Hall, 1101 N. State Street, Rolla, MO 65409-1230

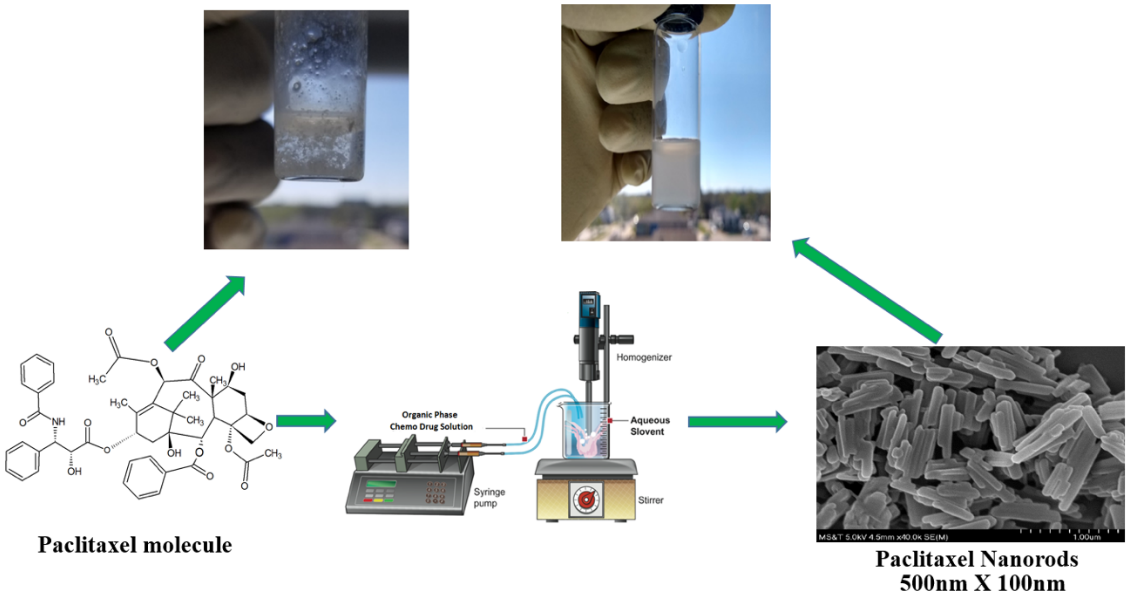


**SI Figure 1: Synthesis process of PTXNRs**. PTXNRs were synthesized using the nanoprecipitation method. Paclitaxel molecules show white floccular aggregation in water, while PTXNRs display complete dispersion in water after the synthesis process.

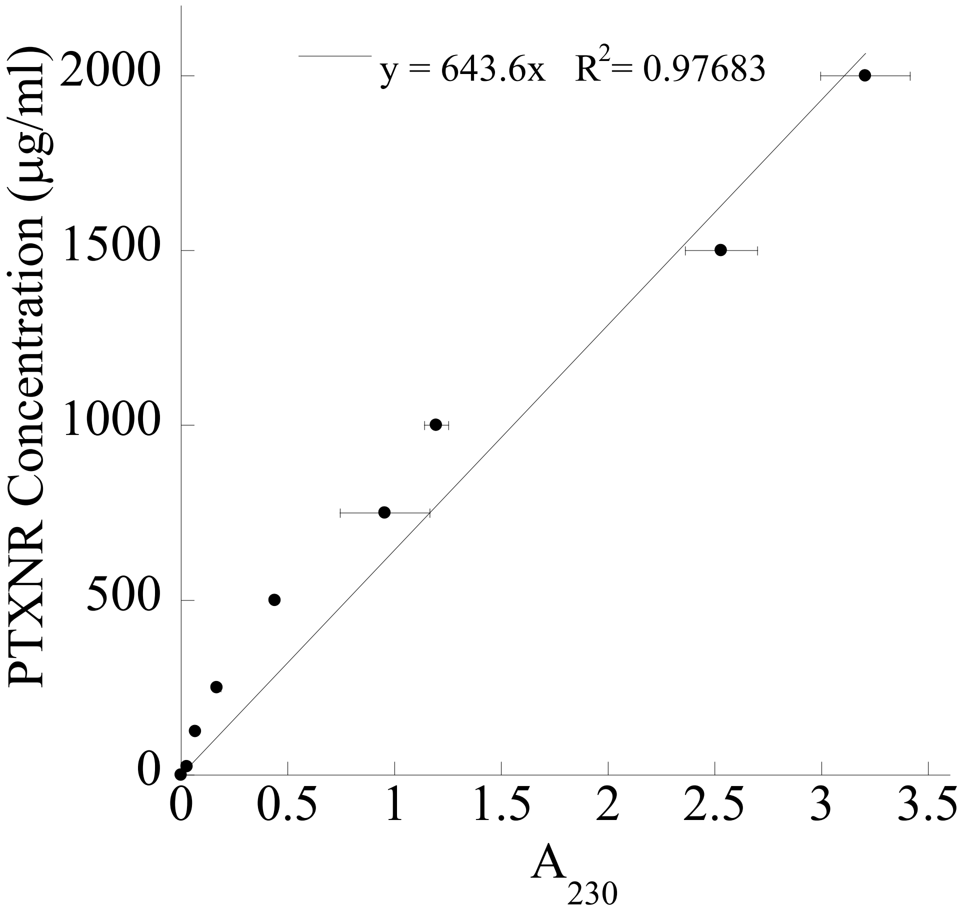


**SI Figure 2:** Calibration curve of PTX for quantification of the amount of PTXNRs in PTXNR-TTZ particles after conjugation with TTZ. The calibration curve was obtained using PTXNRs in PBS at 230 nm absorbance values.

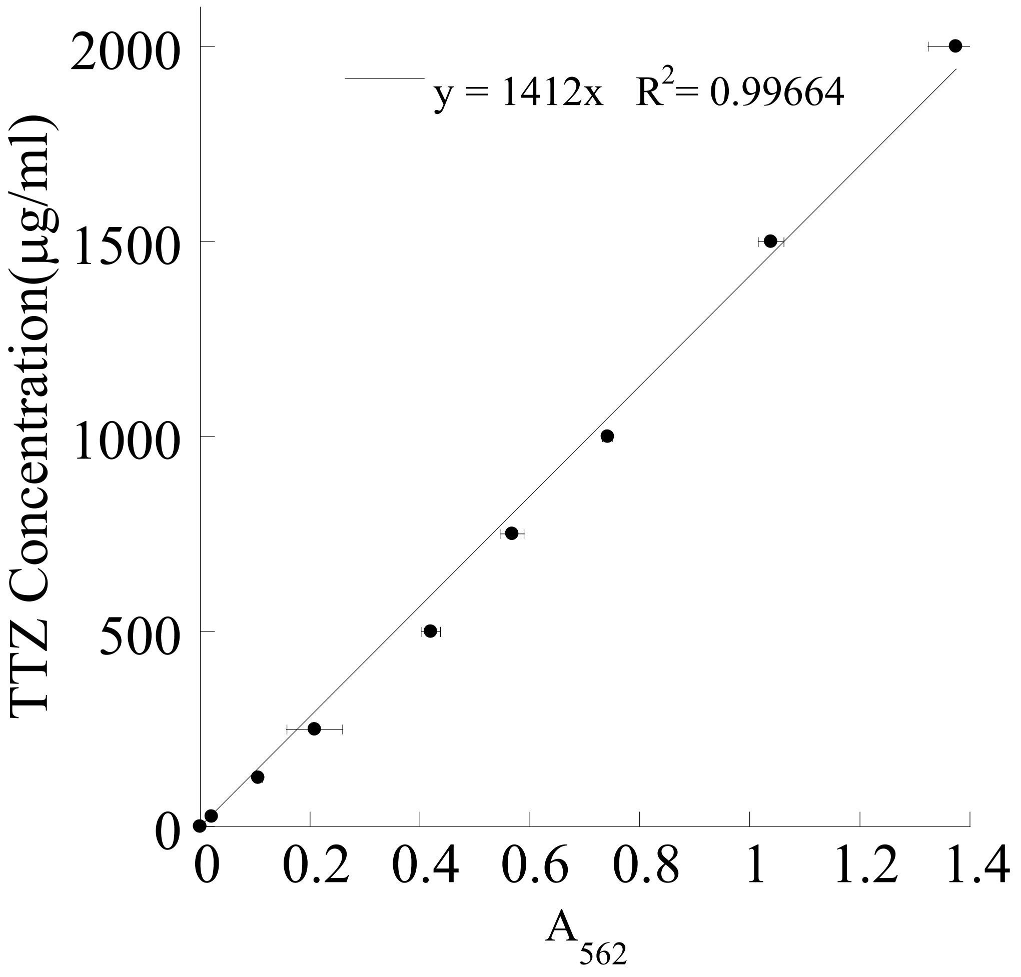


**SI Figure 3:** Calibration curve of Trastuzumab (TTZ) monoclonal antibody for quantification of unconjugated TTZ after conjugation with Paclitaxel nanorods (PTXNRs). The calibration curve was obtained using a BCA protein quantification assay at 562 nm absorbance values.

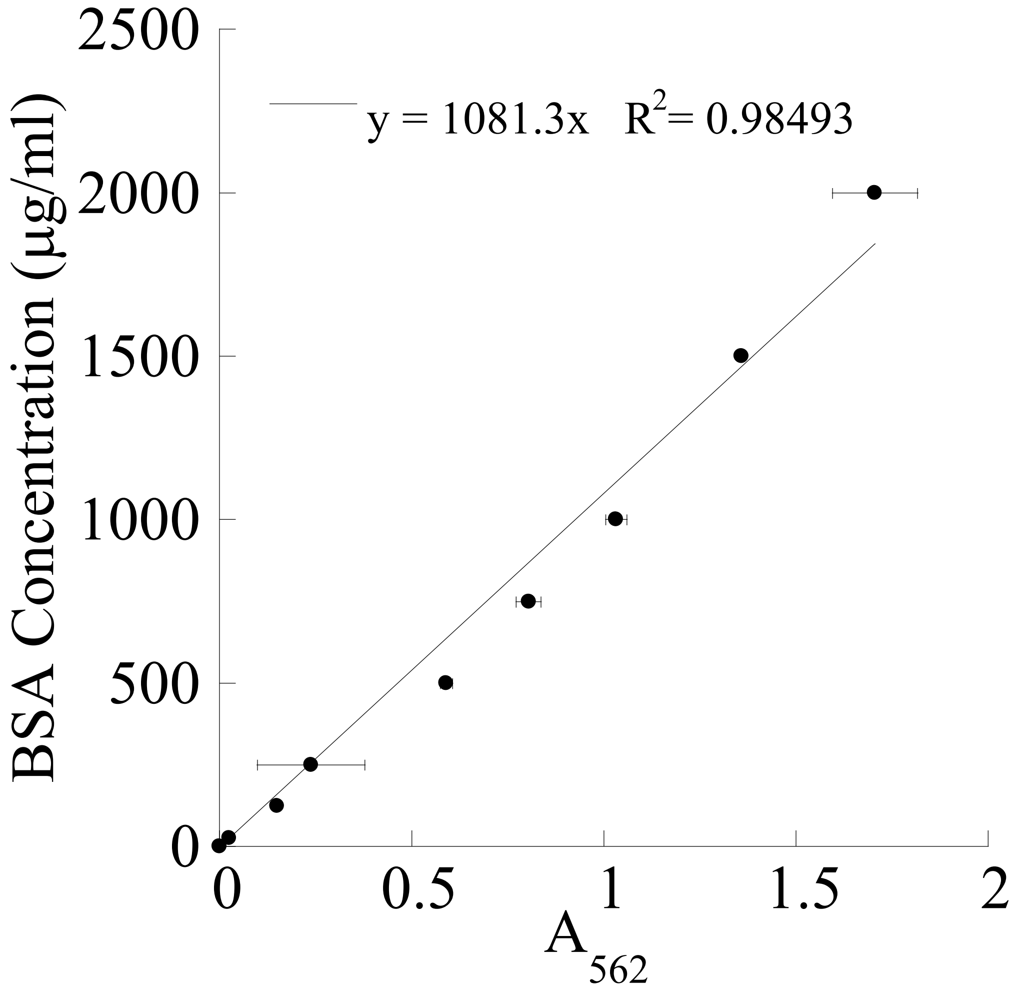


**SI Figure 4:** Calibration curve of BSA protein for quantification of protein in cell lysates for Western blot experiments. The calibration curve was obtained using a BCA protein quantification assay at 562 nm absorbance values.

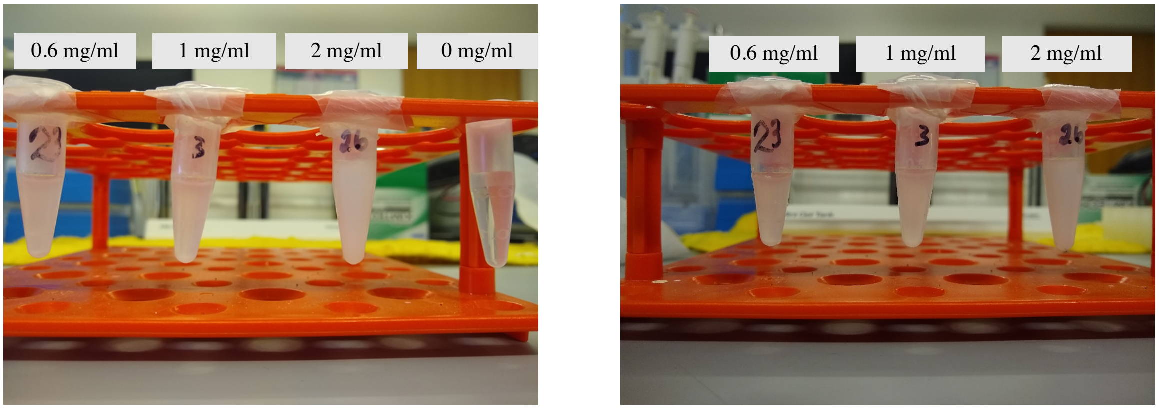


**SI Figure 5:** Colloidal suspensions of PTXNR-TTZ in DI water and PBS at 0.6, 1, and 2 mg/ml concentrations.

**SI Table 1.** Stability analysis of PTXNR and PTXNR-TTZ as a measure of zeta potential value, $\zeta$ (mV)

| | **Zeta potential, $\zeta$ (mV)** | |
|---|---|---|
| **Sample** | **DI water** | **PBS** |
| **PTXNR** | -32.6± 4.82 | -13.4± 2.81 |
| **PTXNR-TTZ** | -17.1±3.83 | -9.5± 0.02 |

**SI Table 2:** Analysis of TTZ conjugation on the surface of PTXNRs

| Initial PTX amount (mg) | % NR formation | Initial PTXNR-CDI amount (mg) | Initial TTZ amount (mg) | % TTZ conjugation efficiency | Amount of PTXNR in 1 mg of PTXNR-TTZ (mg) | Amount of TTZ in 1 mg of PTXNR-TTZ (mg) | PTXNR : TTZ (w/w) (molar ratio) |
|---|---|---|---|---|---|---|---|
| 30 | 64.98±2.37 | 1.0 | 0.10 | 95.24±1.40 | 0.8±0.02 | 0.2±0.02 | 4.0 ± 0.53<br>(681.67 ± 90.23) |

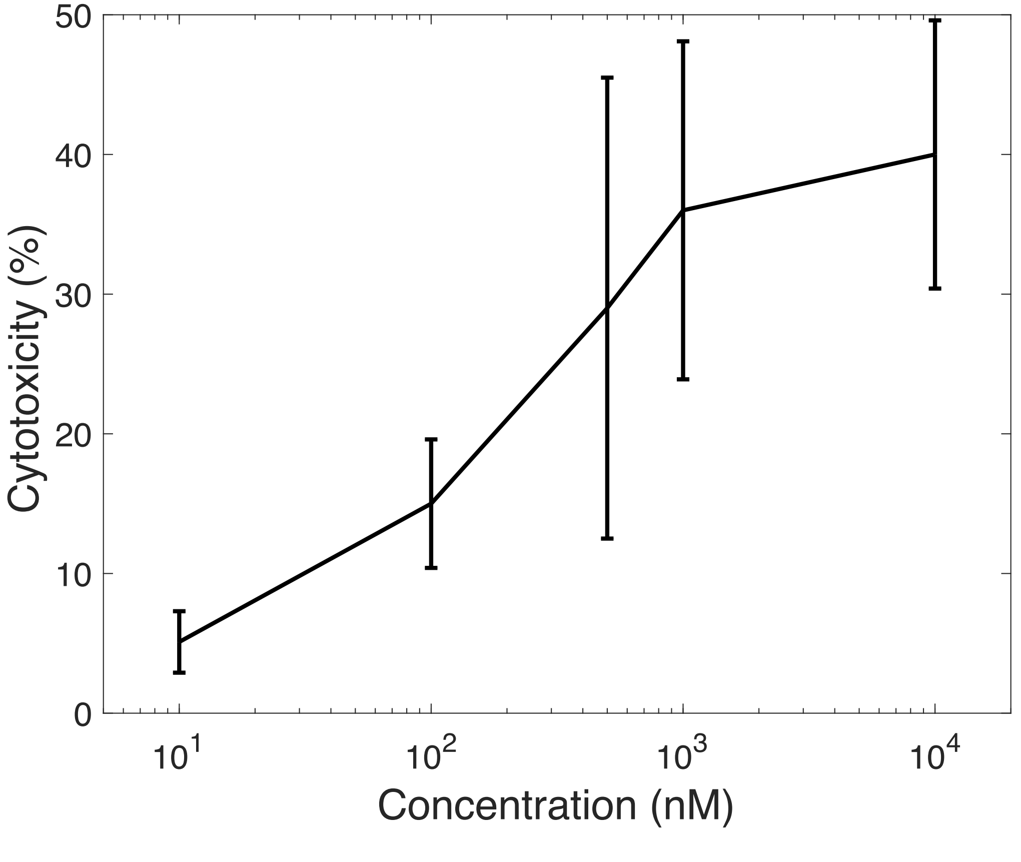


**SI Figure 6:** PTXNR-TTZ showed non-specific cytotoxicity in MCF-10A normal cells *in vitro*.

# SI Table 3: Cytotoxicity Analysis

## BT-474

| | | *p*-value | | | | |
|---|---|---|---|---|---|---|
| Treatment | *vs*. Treatment | **10 nM** | **100 nM** | **500 nM** | **1000 nM** | **10000 nM** |
| PTXNR-TTZ | PTXNR | 0.8206 | 0.0296* | 0.0004* | 0.0007* | <.0001* |
| PTXNR-TTZ | TTZ | 0.9579 | 0.0003* | <.0001* | <.0001* | <.0001* |
| PTXNR-TTZ | PTX solution | 0.2244 | 0.3757 | 0.0003* | 0.0002* | <.0001* |
| PTXNR-TTZ | PTX solution+ TTZ | 0.9677 | 0.0273* | <.0001* | 0.1745 | 0.0019* |
| PTX solution+ TTZ | PTXNR | 0.7809 | <.0001* | 0.6475 | 0.0188* | 0.0743 |
| PTX solution+ TTZ | TTZ | 0.9898 | <.0001* | 0.1194 | <.0001* | <.0001* |
| PTX solution+ TTZ | PTX solution | 0.2241 | 0.1499 | 0.7523 | 0.0055* | 0.0014* |
| PTXNR | TTZ | 0.7712 | 0.0207* | 0.0562 | <.0001* | 0.0031* |
| PTXNR | PTX solution | 0.1495 | 0.0020* | 0.8910 | 0.5452 | 0.0873 |
| PTX solution | TTZ | 0.2280 | <.0001* | 0.0743 | 0.0006* | 0.1651 |

## MDA-MB-231

| | | *p* -value | | | | |
|---|---|---|---|---|---|---|
| Treatment | *vs*. Treatment | **10 nM** | **100 nM** | **500 nM** | **1000 nM** | **10000 nM** |
| PTXNR-TTZ | PTXNR | 0.1170 | 0.0002* | 0.0186* | 0.0246* | 0.0775 |
| PTXNR-TTZ | TTZ | 0.0750 | <.0001* | <.0001* | <.0001* | 0.0002* |
| PTXNR-TTZ | PTX solution | 0.2051 | 0.7412 | 0.4026 | 0.3234 | 0.8855 |
| PTXNR-TTZ | PTX solution+ TTZ | 0.9123 | <.0001* | 0.0031* | <.0001* | <.0001* |
| PTX solution+ TTZ | PTXNR | 0.1282 | <.0001* | <.0001* | <.0001* | <.0001* |
| PTX solution+ TTZ | TTZ | 0.0811 | <.0001* | <.0001* | <.0001* | <.0001* |
| PTX solution+ TTZ | PTX solution | 0.1562 | <.0001* | 0.0005* | 0.0009* | <.0001* |
| PTXNR | TTZ | 0.8651 | <.0001* | <.0001* | <.0001* | 0.0152* |
| PTXNR | PTX solution | 0.0070* | 0.0004* | 0.1434 | 0.0032* | 0.1228 |
| PTX solution | TTZ | 0.0070* | <.0001* | <.0001* | <.0001* | 0.0004* |

## SK-BR-3

| | | *p* -value | | | | |
|---|---|---|---|---|---|---|
| Treatment | *vs*. Treatment | **10 nM** | **100 nM** | **500 nM** | **1000 nM** | **10000 nM** |
| PTXNR-TTZ | PTXNR | <.0001* | 0.8554 | 0.6319 | 0.2213 | 0.0920 |
| PTXNR-TTZ | TTZ | <.0001* | <.0001* | <.0001* | <.0001* | <.0001* |
| PTXNR-TTZ | PTX solution | 0.5424 | 0.0941 | 0.5570 | 0.1245 | <.0001* |
| PTXNR-TTZ | PTX solution+ TTZ | 0.8684 | 0.6750 | 0.5804 | 0.0776 | 0.0011* |
| PTX solution+ TTZ | PTXNR | <.0001* | 0.7713 | 0.9276 | 0.4897 | 0.0286* |
| PTX solution+ TTZ | TTZ | <.0001* | <.0001* | <.0001* | <.0001* | <.0001* |
| PTX solution+ TTZ | PTX solution | 0.3447 | 0.1213 | 0.9661 | 0.8003 | 0.2079 |
| PTXNR | TTZ | 0.9758 | <.0001* | <.0001* | <.0001* | <.0001* |
| PTXNR | PTX solution | <.0001* | 0.0680 | 0.8939 | 0.6738 | 0.0011* |
| PTX solution | TTZ | <.0001* | <.0001* | <.0001* | <.0001* | <.0001* |

# SI Table 4: Apoptosis Assay (Annexin-V-FITC/PI)

## BT-474

| | | *p* -value | | | | | | | | | | | |
|---|---|---|---|---|---|---|---|---|---|---|---|---|---|
| Treatment | *vs.* Treatment | **Early apoptotic (Annexin-V-FITC)** | | | | **Non-apoptotic dead (PI)** | | | | **Late apoptotic dead (Annexin-V-FITC/PI)** | | | |
| | | **0 h** | **24 h** | **48 h** | **72 h** | **0 h** | **24 h** | **48 h** | **72 h** | **0 h** | **24 h** | **48 h** | **72 h** |
| PTXN R-TTZ | Untreated | 0.6536 | 0.0257* | 0.0017* | <.0001* | 0.5569 | <.0001* | 0.0003* | 0.6488 | 0.0831 | 0.1321 | 0.0005* | 0.0003* |

## MDA-MB-231

| | | *p* -value | | | | | | | | | | | |
|---|---|---|---|---|---|---|---|---|---|---|---|---|---|
| Treatment | *vs.* Treatment | **Early apoptotic (Annexin-V-FITC)** | | | | **Non-apoptotic dead (PI)** | | | | **Late apoptotic dead (Annexin-V-FITC/PI)** | | | |
| | | **0 h** | **24 h** | **48 h** | **72 h** | **0 h** | **24 h** | **48 h** | **72 h** | **0 h** | **24 h** | **48 h** | **72 h** |
| PTXN R-TTZ | Untreated | 0.6624 | 0.4486 | 0.0082* | 0.0407* | 0.0037* | 0.0008* | 0.0002* | 0.0144* | 0.0064* | 0.0104* | <.0001* | 0.0429* |

## SK-BR-3

| | | *p* -value | | | | | | | | | | | |
|---|---|---|---|---|---|---|---|---|---|---|---|---|---|
| Treatment | *vs.* Treatment | **Early apoptotic (Annexin-V-FITC)** | | | | **Non-apoptotic dead (PI)** | | | | **Late apoptotic dead (Annexin-V-FITC/PI)** | | | |
| | | **0 h** | **24 h** | **48 h** | **72 h** | **0 h** | **24 h** | **48 h** | **72 h** | **0 h** | **24 h** | **48 h** | **72 h** |
| PTXN R-TTZ | Untreated | - | 0.1871 | 0.2947 | 0.3135 | 0.4910 | 0.0553 | 0.0295* | 0.5938 | 0.1957 | 0.0964 | 0.0002* | 0.0003* |

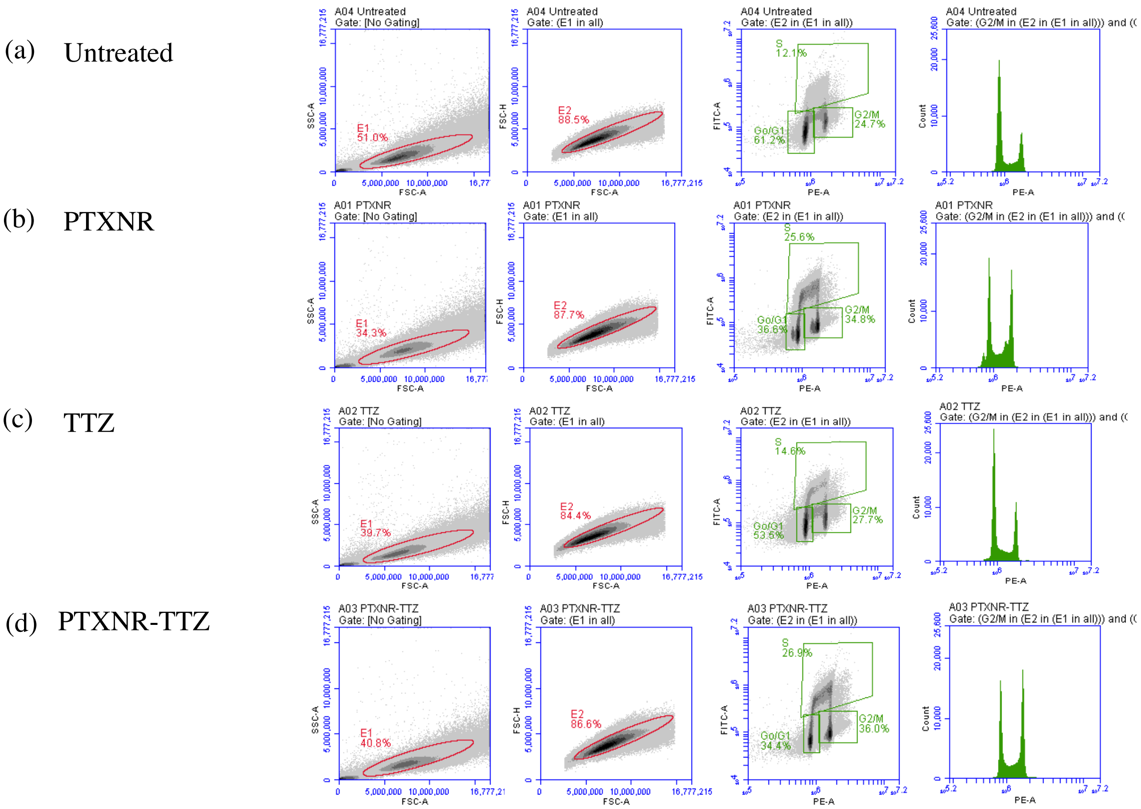


**SI Figure 7:** Flow cytometry data for cell cycle analysis of **(a)** untreated, **(b)** PTXNR, **(c)** TTZ, and **(d)** PTXNR-TTZ treated BT-474 cells after 24 h. The side scatter (SSC-A) *versus* forward scatter (FSC-A) gating is used to identify BT-474 cells as gated by E1. The forward scatter height (FSC-H) *versus* forward scatter area (FSC-A) density plots exclude doublet cells for accurate counting as shown by E2 gating. The plots showing FITC-A and PE-A represent live cells and their stained DNA, respectively, and gated for DNA analysis using Count *versus* PE-A plots. The Count *versus* PE-A plots were analyzed using the flow cytometry software to calculate the percentage arrests in G0/G1 (first sharp peak), S (flattened areas), and G2/M (second sharp peak) phases.

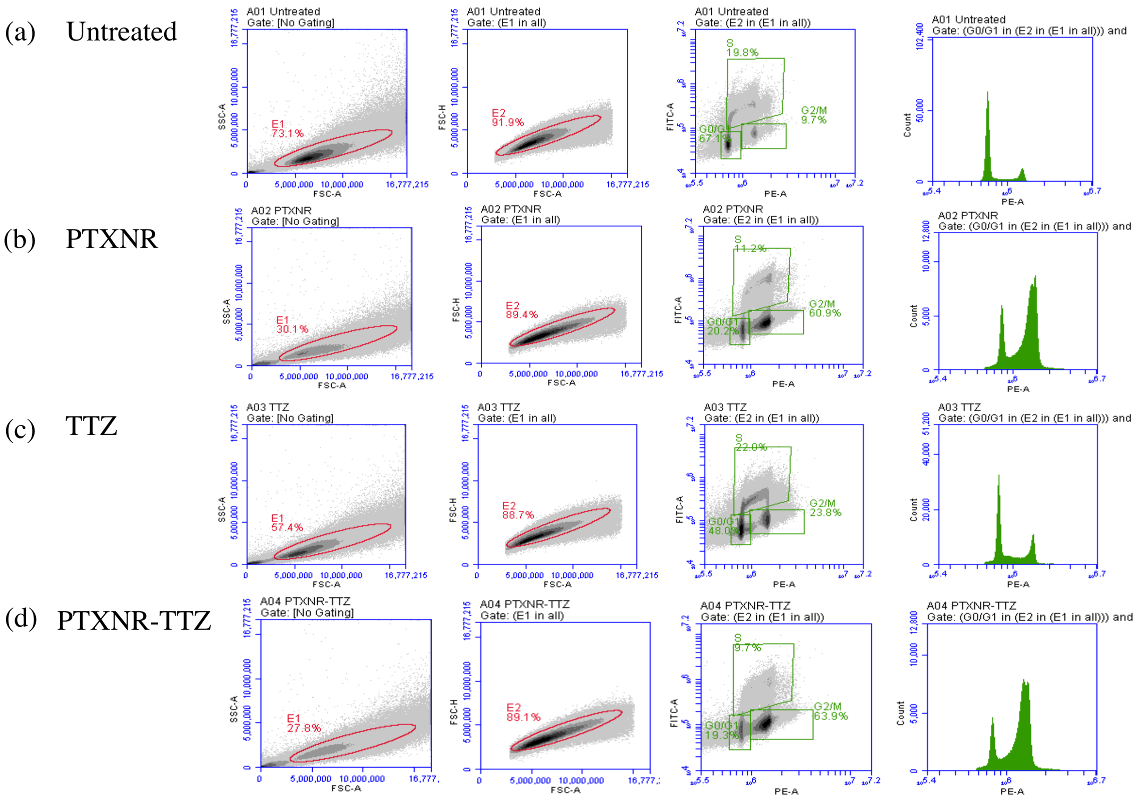


**SI Figure 8:** Flow cytometry data for cell cycle analysis of **(a)** untreated, **(b)** PTXNR, **(c)** TTZ, and **(d)** PTXNR-TTZ treated BT-474 cells after 48 h. The histogram of relative DNA content in Count *versus* PE-A plots shows a shift in peaks from G0/G1 to G2/M cells in PTXNR and PTXNR-TTZ treated cells compared to untreated and TTZ treated cells.

(a) Untreated

(b) PTXNR

(c) TTZ

(d) PTXNR-TTZ

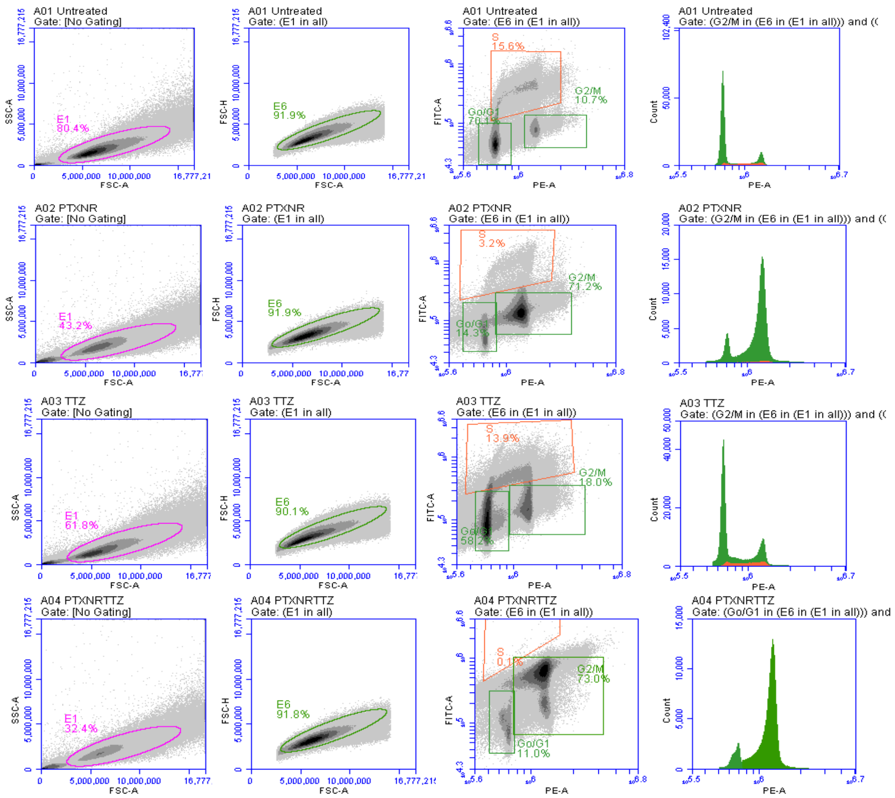


**SI Figure 9:** Flow cytometry data for cell cycle analysis of **(a)** untreated, **(b)** PTXNR, **(c)** TTZ, and **(d)** PTXNR-TTZ treated BT-474 cells after 72h. By the end of the incubation periods, >80% of cells were arrested in G2/M phases after PTXNR and PTXNR-TTZ treatments, while untreated and TTZ treated cells showed most cells in G0/G1.

# SI Table 5: Cell Cycle Analysis

## BT-474

| | | *p* -value | | | | | | | | |
|---|---|---|---|---|---|---|---|---|---|---|
| Treatment | *vs.* Treatment | **24 h** | | | **48 h** | | | **72 h** | | |
| | | **G0/G1** | **S** | **G2/M** | **G0/G1** | **S** | **G2/M** | **G0/G1** | **S** | **G2/M** |
| PTXNR-TTZ | Untreated | 0.0551 | 0.5392 | 0.0382* | 0.0029* | 0.2282 | 0.0017* | 0.0005* | 0.0021* | 0.0007* |
| PTXNR-TTZ | PTXNR | 0.8429 | 0.8978 | 0.9780 | 0.8852 | 0.6973 | 0.7662 | 0.6049 | 0.1159 | 0.3776 |
| PTXNR-TTZ | TTZ | 0.2262 | 0.6231 | 0.3015 | 0.0245* | 0.4362 | 0.0139* | 0.0014* | 0.0051* | 0.0018* |
| PTXNR | Untreated | 0.0689 | 0.6218 | 0.0393* | 0.0027* | 0.3725 | 0.0020* | 0.0006* | 0.0072* | 0.0010* |
| PTXNR | TTZ | 0.2902 | 0.7131 | 0.3121 | 0.0214* | 0.6787 | 0.0181* | 0.0018* | 0.0238* | 0.0032* |
| TTZ | Untreated | 0.2786 | 0.8962 | 0.1364 | 0.0408* | 0.6070 | 0.0286* | 0.0646 | 0.2088 | 0.0858 |

# SI Table 6: Western Blot Analysis

## BT-474

| | | *p*-value | | | | | |
|---|---|---|---|---|---|---|---|
| Treatment | *vs.* Treatment | **XIAP** | **Actin** | **Cleaved Caspase - 9 (p10)** | **Caspase 3** | **Cleaved Caspase -3** | **Cyt- C** |
| PTXNR-TTZ | Untreated | 0.0094* | <.0001* | 0.0235* | 0.0150* | 0.0494* | 0.0391* |
| PTXNR-TTZ | PTXNR | 0.3926 | 0.0721 | 0.3251 | 0.4507 | 0.7168 | 0.7549 |
| PTXNR-TTZ | TTZ | 0.0243* | <.0001* | 0.0693 | 0.0099* | 0.4133 | 0.5425 |
| PTXNR | Untreated | 0.0375* | <.0001* | 0.0094* | 0.0622 | 0.0301* | 0.0548 |
| PTXNR | TTZ | 0.0989 | <.0001* | 0.0230* | 0.0417* | 0.6332 | 0.7576 |
| TTZ | Untreated | 0.5503 | 0.6209 | 0.3330 | 0.8262 | 0.0163* | 0.0779 |

## MDA-MB-231

| | | *p* -value | | | | | |
|---|---|---|---|---|---|---|---|
| Treatment | *vs.* Treatment | **XIAP** | **Actin** | **Cleaved Caspase - 9 (p10)** | **Caspase 3** | **Cleaved Caspase -3** | **Cyt- C** |
| PTXNR-TTZ | Untreated | 0.5631 | 0.0028* | 0.7904 | 0.4751 | - | 0.0160* |
| PTXNR-TTZ | PTXNR | 0.1208 | 0.0009* | 0.5113 | 0.3466 | - | 0.3400 |
| PTXNR-TTZ | TTZ | 0.2954 | 0.0046* | 0.3973 | 0.4565 | - | 0.7792 |
| PTXNR | Untreated | 0.2525 | 0.0919 | 0.6854 | 0.8126 | - | 0.0429* |
| PTXNR | TTZ | 0.4880 | 0.0393* | 0.8317 | 0.1058 | - | 0.4777 |
| TTZ | Untreated | 0.5973 | 0.4643 | 0.5437 | 0.1578 | - | 0.0207* |

# Full Western Blot Images of BT-474 as shown in Figure 7(a)

**SI Figure 10 (a):** A single full-length Western gel blot was used for probing multiple protein expressions in BT-474 cells. The blot was cut into multiple strips following the protein transfer as revealed in **Figure 7(a)**. Here, the strip containing approximately 40-80 kDa region was imaged.

## XIAP

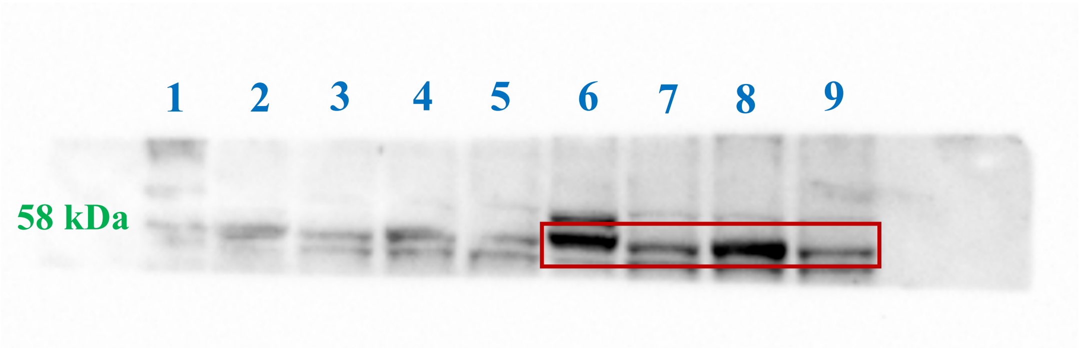


1, Protein ladder; 2, Untreated; 3, PTXNR; 4, TTZ; 5, PTXNR-TTZ; 6, Untreated; 7, PTXNR; 8, TTZ; 9, PTXNR-TTZ

## Actin

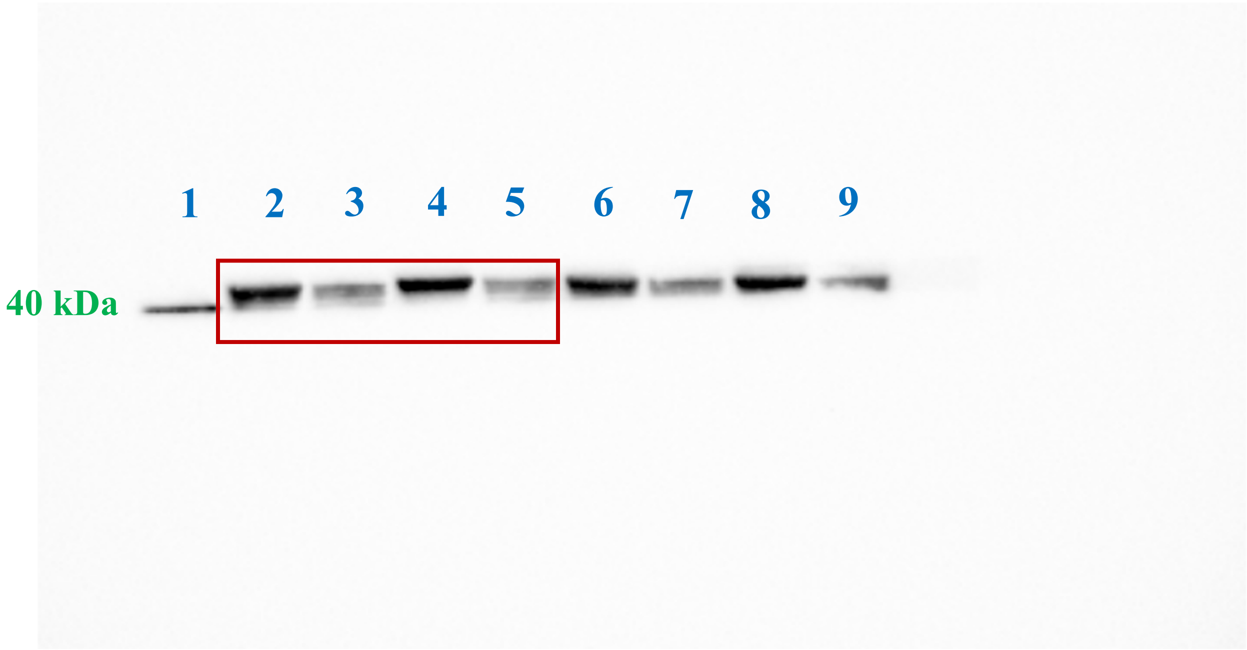


1, Protein ladder; 2, Untreated; 3, PTXNR; 4, TTZ; 5, PTXNR-TTZ; 6, Untreated; 7, PTXNR; 8, TTZ; 9, PTXNR-TTZ

## Fully Cleaved Caspase-9

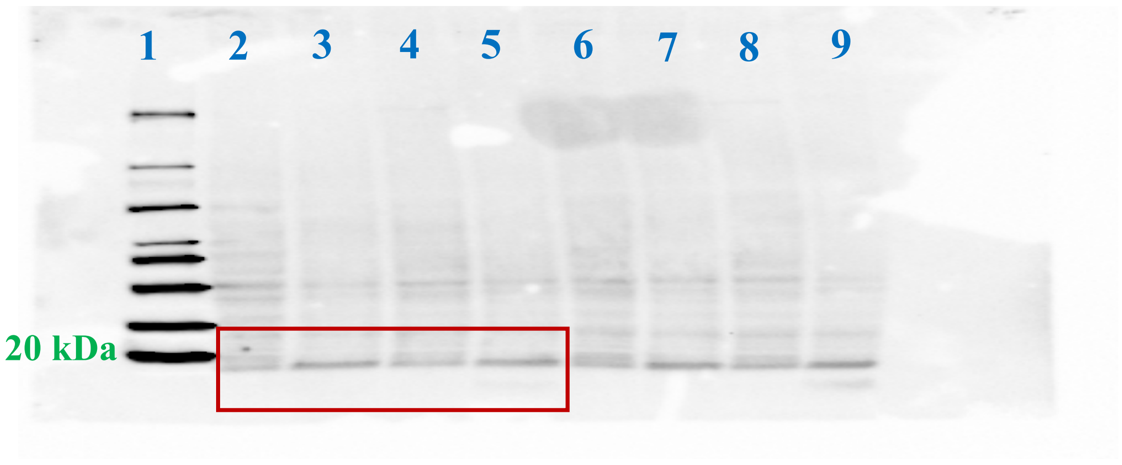


1, Protein ladder; 2, Untreated; 3, PTXNR; 4, TTZ; 5, PTXNR-TTZ; 6, Untreated; 7, PTXNR; 8, TTZ; 9, PTXNR-TTZ

## Caspase-3

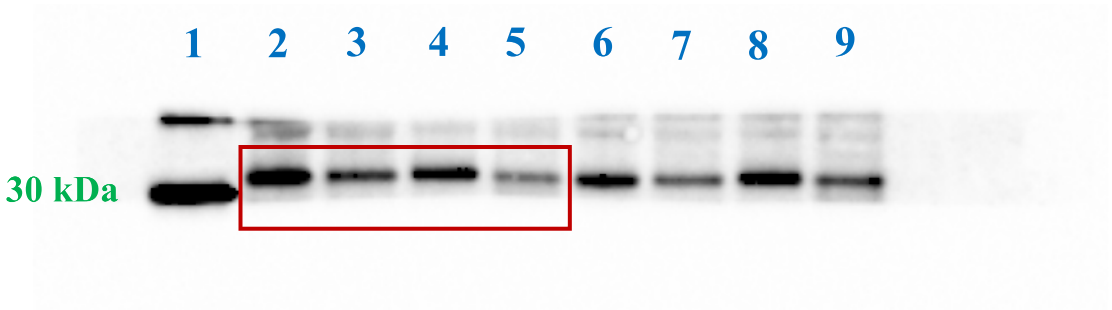


1, Protein ladder; 2, Untreated; 3, PTXNR; 4, TTZ; 5, PTXNR-TTZ; 6, Untreated; 7, PTXNR; 8, TTZ; 9, PTXNR-TTZ. Note: The blot was cut into multiple strips following the protein transfer. A single full-length blot was used for probing multiple protein expression. Here, the strip containing 30-40 kDa region was imaged

## Cleaved Caspase-3

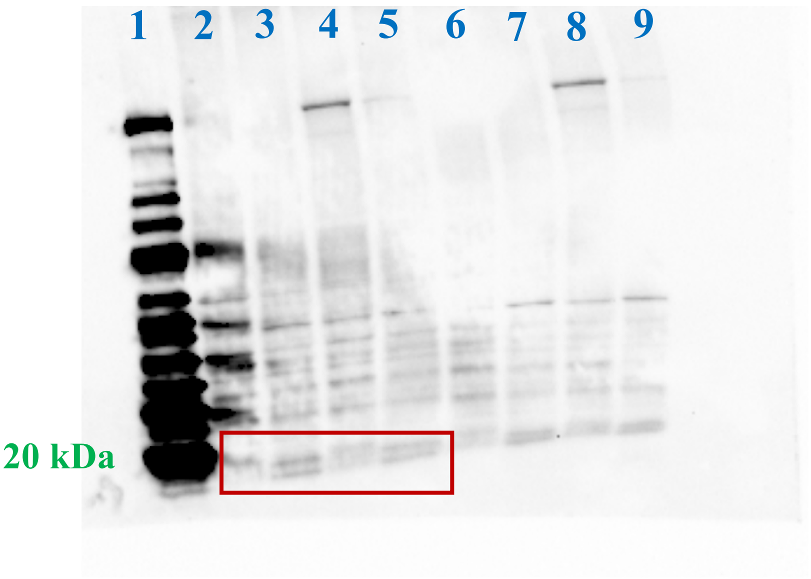


1, Protein ladder; 2, Untreated; 3, PTXNR; 4, TTZ; 5, PTXNR-TTZ; 6, Untreated; 7, PTXNR; 8, TTZ; 9, PTXNR-TTZ

## Cytochrome-C

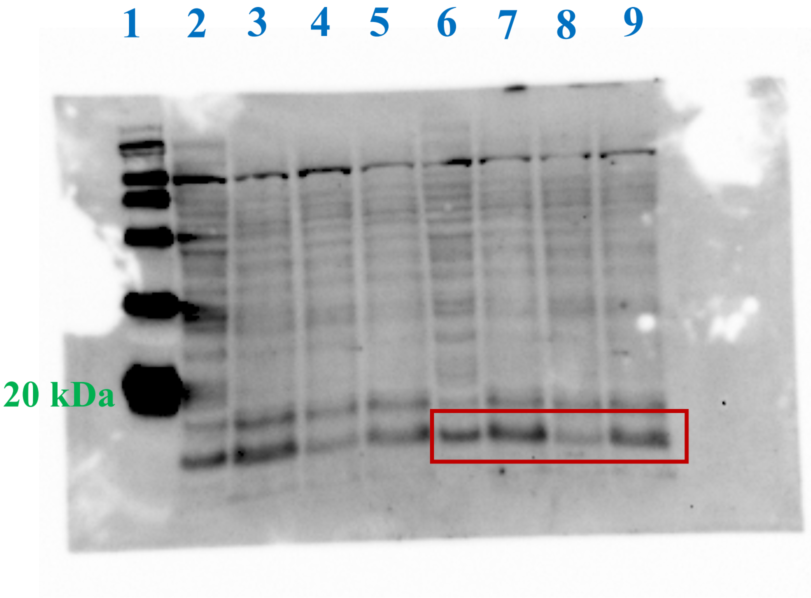

1, Protein ladder; 2, Untreated; 3, PTXNR; 4, TTZ; 5, PTXNR-TTZ; 6, Untreated; 7, PTXNR; 8, TTZ; 9, PTXNR-TTZ

# <u>GAPDH</u>

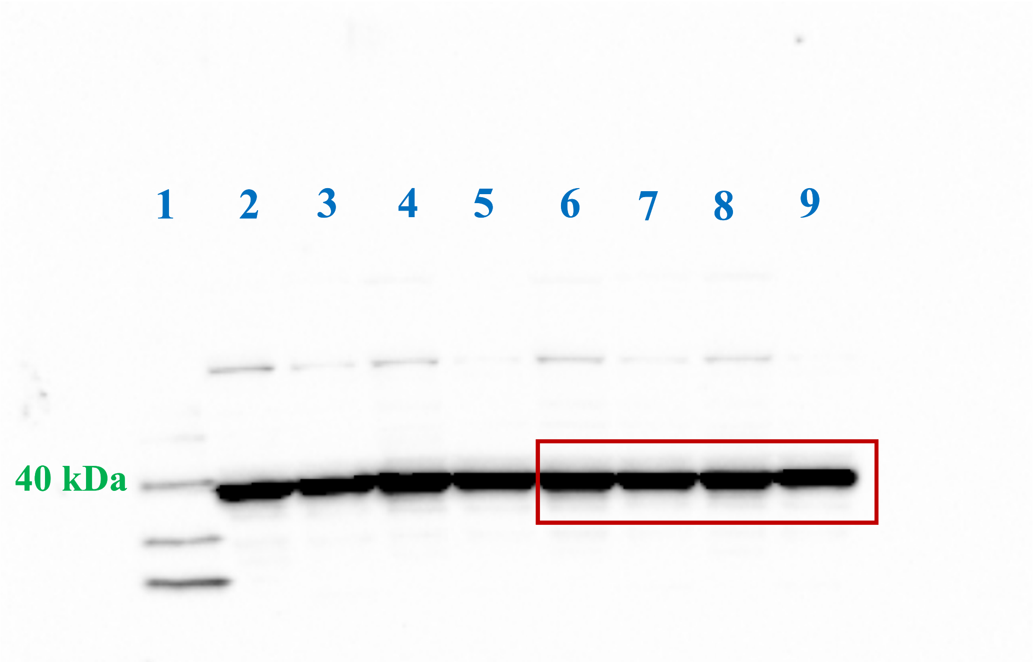


1, Protein ladder; 2, Untreated; 3, PTXNR; 4, TTZ; 5, PTXNR-TTZ; 6, Untreated; 7, PTXNR; 8, TTZ; 9, PTXNR-TTZ

# Full Western Blot Images of MDA-MB-231 as shown in Figure 7(b)

**SI Figure 10 (b):** A single full-length blot was used for probing multiple protein expressions in MDA-MB-231 cells. The blot was cut into multiple strips following the protein transfer. Here, the strip containing 40-80 kDa region was imaged as revealed in **Figure 7(b)**.

## XIAP

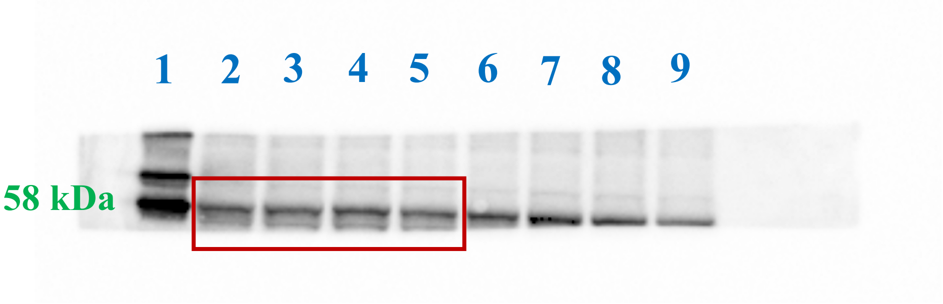


1, Protein ladder; 2, Untreated; 3, PTXNR; 4, TTZ; 5, PTXNR-TTZ; 6, Untreated; 7, PTXNR; 8, TTZ; 9, PTXNR-TTZ

## Actin

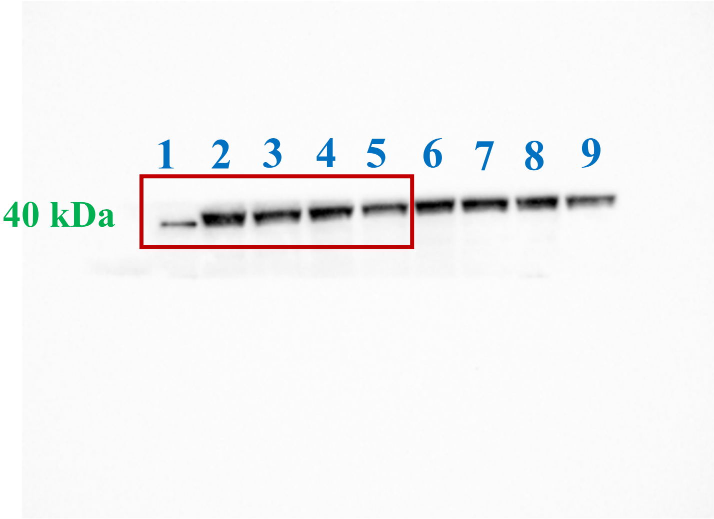


1, Protein ladder; 2, Untreated; 3, PTXNR; 4, TTZ; 5, PTXNR-TTZ; 6, Untreated; 7, PTXNR; 8, TTZ; 9, PTXNR-TTZ

## Fully Cleaved Caspase-9

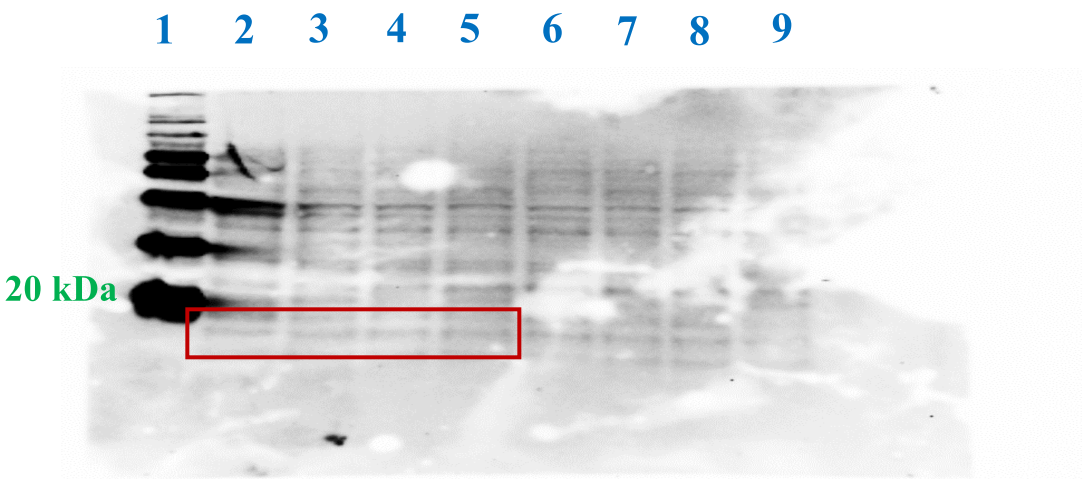


1, Protein ladder; 2, Untreated; 3, PTXNR; 4, TTZ; 5, PTXNR-TTZ; 6, Untreated; 7, PTXNR; 8, TTZ; 9, PTXNR-TTZ

## Caspase-3

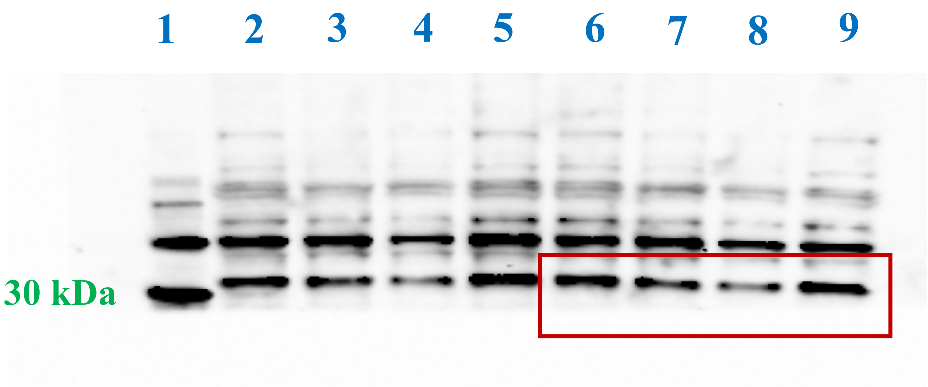


1, Protein ladder; 2, Untreated; 3, PTXNR; 4, TTZ; 5, PTXNR-TTZ; 6, Untreated; 7, PTXNR; 8, TTZ; 9, PTXNR-TTZ

# Cleaved Caspase-3

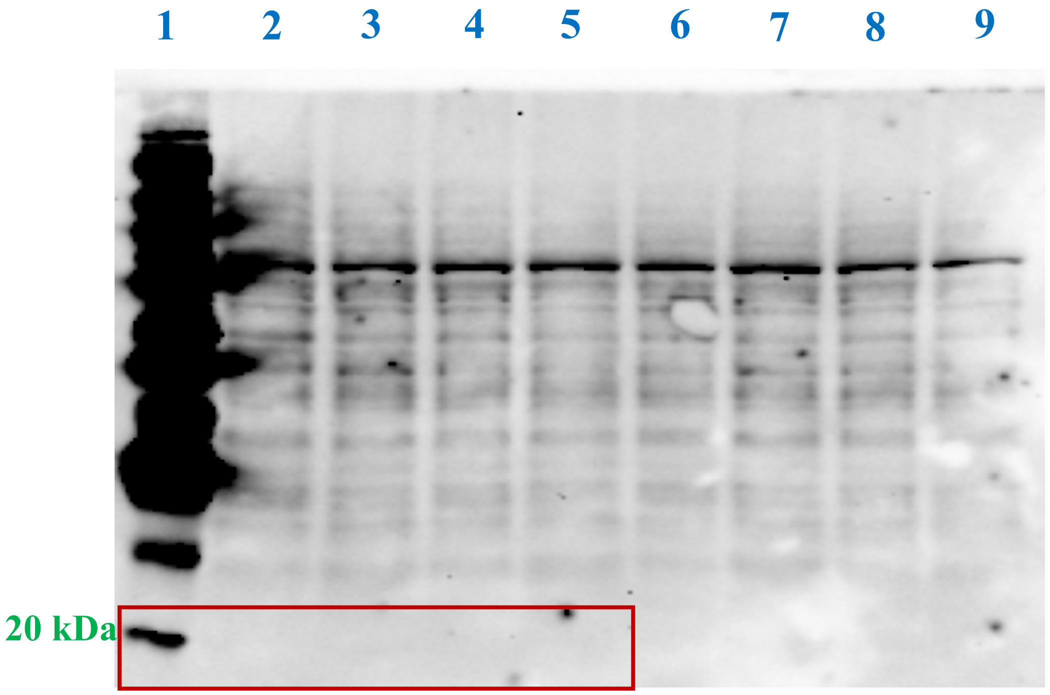


1, Protein ladder; 2, Untreated; 3, PTXNR; 4, TTZ; 5, PTXNR-TTZ; 6, Untreated; 7, PTXNR; 8, TTZ; 9, PTXNR-TTZ

# Cytochrome-C

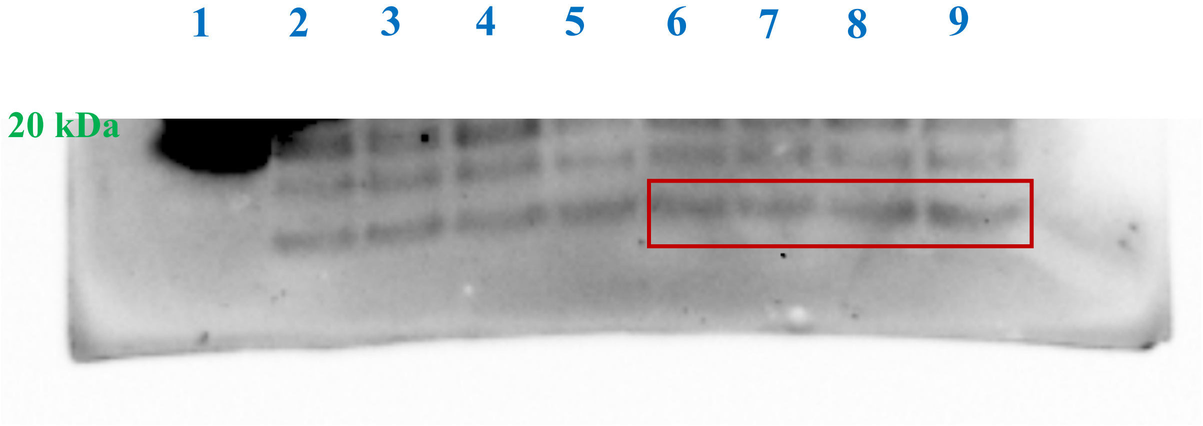


1, Protein ladder; 2, Untreated; 3, PTXNR; 4, TTZ; 5, PTXNR-TTZ; 6, Untreated; 7, PTXNR; 8, TTZ; 9, PTXNR-TTZ. Note: The blot was cut into multiple strips following the protein transfer.

A single full-length blot was used for probing multiple protein expression. Here, a narrow strip around >20 kDa region was imaged

# GAPDH

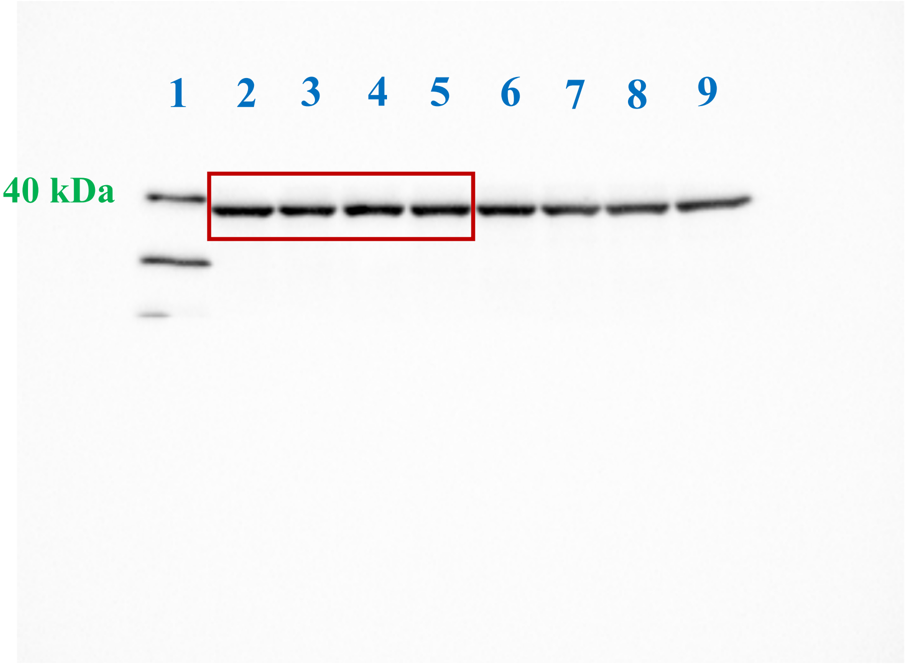


1, Protein ladder; 2, Untreated; 3, PTXNR; 4, TTZ; 5, PTXNR-TTZ; 6, Untreated; 7, PTXNR; 8, TTZ; 9, PTXNR-TTZ
